# Drug-induced cytotoxicity prediction in muscle cells, an application of the Cell Painting assay

**DOI:** 10.1371/journal.pone.0320040

**Published:** 2025-03-31

**Authors:** Roman Lambert, Eva Serrano Candelas, Pablo Aparicio, Aisling Murphy, Rafael Gozalbes, Howard Oliver Fearnhead

**Affiliations:** 1 University of Galway, School of Medicine, Pharmacology & Therapeutics, Galway, Ireland; 2 ProtoQSAR SL, CEEI Parque Tecnológico de Valencia, Paterna, Spain; 3 Moldrug AI Systems SL, Valencia, Spain; Huashan Hospital Fudan University, CHINA

## Abstract

*In silico* toxicity prediction offers the chance of reducing or replacing most animal testing through the integration of large experimental assay datasets with the appropriate computational approaches. The use of Cell Painting to detect various phenotypic changes induced by chemicals is emerging as a powerful technique in toxicity prediction. However, most Cell Painting approaches use cancer cells that are less relevant for many toxicological endpoints, which may limit the usefulness of this data. In this study, a myoblast cell line is used to characterize cellular responses to a panel of 30 known myotoxicants. In place of traditional structural descriptors, here each perturbation is described by a fingerprint of calculated properties, deducted from the intensity, shape, or texture of individual cells. We show that these kinds of descriptors convey information to allow the prediction of the cellular viability and fate of cells in myoblasts and differentiated myotubes of the C2C12 cell line, and the clustering of drugs by their cytotoxicity responses.

## Introduction

Various venoms, drugs, toxins, or environmental chemicals cause muscle toxicity, resulting in muscle damage, inflammation, pain, and in rare cases death (reviewed in [1]). Thus, structurally diverse chemicals with a range of molecular targets can induce myotoxicity [2]. However, although over 200 drugs and chemicals are myotoxic, toxicology has focussed on other important target organs such as the liver (hepatotoxicity), kidney (nephrotoxicity), and nervous system (neurotoxicity), as highlighted by the number of article published in each subfield of toxicology, as seen in S2 Fig. Economic and ethical reasons have driven the development of in vitro toxicology approaches for these target organs to allow rapid identification of toxic hazards and for establishing toxic levels (reviewed in [3]). In contrast, although there are cell culture models for studying muscle cell biology and pathology, they have rarely been used in a toxicological context.

Reasons why this area of toxicology has received less attention include myotoxicity often being a secondary effect to other types of toxicity (e.g., hepatotoxicity) [4], difficulties in diagnosing myotoxicity [1] and myotoxicity being perceived as a relative mild toxicity, even though it may be long-lasting, or in rare cases fatal [5]. Nonetheless, examples like the global withdrawal of Bayer’s lipid-lowering drug cerivastatin (CERI) after at least 52 cases of fatal rhabdomyolysis [5] indicate the need for increased understanding of myotoxicity. The controversy around the myotoxicity of this compound started in late 1999, when Bayer added a contraindication to avoid while on CERI therapy [5]. Rhabdomyolysis is an adverse reaction expected with all members of the statin family, but despite similar structures and mechanism of pharmacological action the risk with CERI was estimated as 10 times higher than with others statins and was also associated with the concomitant use of gemfibrozil [6]. There are other examples of drug-induced myotoxicity either stopping drug development or inducing significant unwanted effects for important drugs. For example, the phase 3 clinical trial of clevudine, a DNA synthesis inhibitor designed for treating Hepatitis B, was stopped in 2009 because of mild to moderate myopathy [7]. Zidovudine (ZIDO), a nucleoside analogue that is an important component of HIV-AIDS treatment, triggers a type of mitochondrial myopathy in up to 20% of patients, depending on the duration of treatment [8]. Besides drugs, Germanium, which has been found in dietary supplements and other types of “over the counter products” can cause myopathy and MERRF syndrome [9].

Given these issues, there is a clear need to improve our understanding and detection of muscle toxicity by developing (1) the use of in vitro muscle models in toxicology; (2) biomarkers to aid in early diagnosis and monitoring of the condition; (3) better risk assessment that considers the long-term effects of myotoxicity.

The Cell Painting assay (CPA) is a high-throughput microscopy experiment designed for an extensive evaluation of phenotypic features in fixed cells, using a set of 6 fluorescent biomarkers [10]. It allows the simultaneous staining of different organelles of cells in multiple samples to detect morphological changes induced by chemicals. Analysis of the pattern of changes induced by chemicals reveals information about the toxicity of the chemicals and even suggests the chemicals’ mechanism of action [11]. The applications of the CPA are diverse, and new ones are continually developed [12,13]. Importantly, this approach is independent of chemical structure descriptors and physicochemical properties of the studied chemicals. The C2C12 cell line is a mouse myoblast line [14]. The cells have been extensively used to study both myogenesis and myotoxicity [15–17]. C2C12 cells model a progenitor/stem cell population called satellite cells that are essential for muscle regeneration [18], and can be induced to differentiate into polynucleated myotubes [19]. The cells therefore provide an *in vitro* model to easily study two different cell types that are central to skeletal muscle biology.

Here we investigated the use of the C2C12 muscle cell line to assess high throughput and high content approaches previously used in other toxicology models [11,20,21] to detect muscle toxicants. The specific aims were to assess if the approach can detect known muscle toxicants, discriminate between toxicants with different mechanisms of action and detect concentration-dependent changes that could be used to determine points of departure. This is the first cell painting study using C2C12 cells and the first comparing myoblasts and myotubes. As such it is the foundation for future studies to improve in vitro detection of myotoxicants.

## Results

### Cell Painting imaging and phenotyping

**Fig 1:**
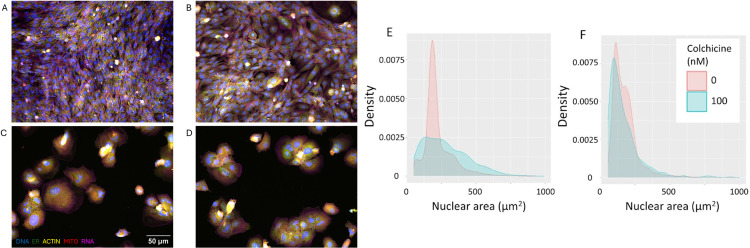
Structural changes in C2C12 myoblasts treated with colchicine. Composite fluorescence microscopy images captured after a 72 h treatment with DMSO 0.1% as vehicle control (A), or COLC at various concentrations: 10 nM (B), 3 µ M (C), 30 µ M (D). 20X magnification, scale bar of 50 µ M for reference. Channel color mapping: blue - DNA, red - Mitochondria, green - ER, magenta - Actin, yellow - RNA. Contrast adjusted on DNA channel for improved readability. (E) The density of nuclear area (µm^2^) of control and Colchicine (100 nM) treated myoblasts and (F) myotubes.

The CPA revealed a range of morphological and other perturbations induced by toxicant treatment. Phenotypic changes included increased nucleus and cell size, decreased count, cells gathered in groups, formation of multinucleated cells, and important rearrangements in the actin cytoskeleton structure. Our expectation was that the data would include drug-specific, cell-type specific and concentration dependent changes. Colchicine (Fig 1 and S1 Fig) and etoposide (S1 Fig) are examples of drugs inducing drug specific, cell-type specific and concentration dependent phenotypic changes. Nuclear size (assessed by measuring nuclear area) is a cell feature that is affected by these two drugs because of their respective molecular targets and mechanism of action. Even at a concentration as low as 100 nM, the lowest dose at which a significant decrease of cell count was observed, the entire cell population treated with COLC undergoes a drastic morphological change, as pictured in Fig 1B. Our ability to detect the predicted effects of colchicine and etoposide on nuclear area prompted us to go further and analyze the complete data set.

All the collected images were then processed following an automated protocol, which gave us quantified single-cell phenotypic data for each perturbation, including features describing morphology, intensity, symmetry, and texture properties, totaling 1070 features extracted with Harmony and around 1700 extracted with CellProfiler. A description of feature extraction protocols can be found in S1 Table. Our CellProfiler pipeline is adapted from the JUMP CPA pipeline for feature calculation, with several tweaks and optimizations, such as typical cell size to match the C2C12 specifications. The number and the diversity of these toxic phenotypes pushed us to find the optimal way to quantify the changes happening in the cell morphology, starting for instance with the calculation of cell count from these CPA images. Optimal phenotypes were obtained using the Robust MAD (Median Absolute Deviation) normalization method, as previously recommended by the Carpenter-Singh lab directives and tends to be more popular than a traditional Z-score scaling in recent studies [11,13,22–24]. These resulting phenotypes were used for further analysis.

### Cell counting from Cell Painting image segmentation

**Fig 2:**
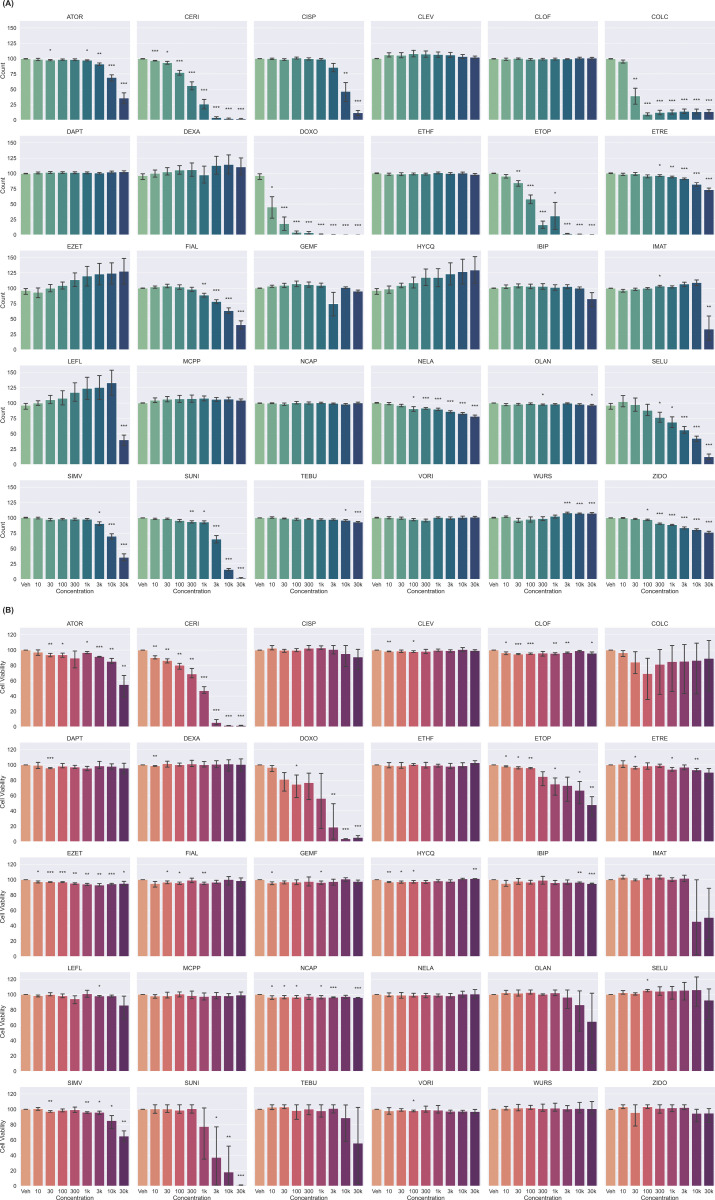
Median cell counts per well (A) and median cell viability (B) plotted against 8 concentrations for the 30 compounds of the Toxifate library in C2C12 myoblasts treatments. Results are shown as median value ±  standard error from three biological replicates and four technical replicates, n = 12 (3 different plates plated on different days). Statistical significance was assessed by independent T-tests against the mean of vehicle controls for each motherplate. Meaning of symbols: * : p-value <  0.05, **: p-value <  0.01, ***: p-value <  0.001, no symbol: ns.

Seventeen of the 30 compounds caused a significant decrease in median cell count compared to DMSO controls, as seen in Fig 2A. Clear concentration-dependent effects were observed with different compounds displaying very different potencies (see LC50 values; S8 Table). Detectable cells were close to zero in wells treated with 30 µ M SUNI, ETOP, CERI and DOXO, suggesting that no cell survived at this concentration, or that their shape and size are not comparable to regular healthy cells. To differentiate between these two possibilities (cells were all dead or cells were present but not detectable by CPA), a cell viability assay was performed.

### Comparison to cell viability data from an ATP-based assay

Using intracellular ATP measurements from the Cell Titer Glo assay as a surrogate for cell viability is a common approach used to assess toxicant cytotoxicity [25] and is used to derive important points of departure such as NOAEL and LOAEL as well as IC_50_ values that can be valuable in determining the relative toxicity of several compounds.

The most cytotoxic drugs in our panel (in descending order) are CERI, SUNI, DOXO, ATOR, ETOP and IMAT (Fig 2B). Several compounds displayed similar trends between cell number and cell count. Compounds with diverse molecular targets but mechanisms of action that involve cell cycle arrest (CISP, COLC, DOXO and ETOP), as well as SELU all induce a profound decrease in cell count at concentrations lower than the concentration that decrease ATP levels. This is consistent with the idea that induction of cell death is a response to the cell-cycle effects of these compounds.

However, there were also examples of decreased cell count without decreased ATP levels. For instance colchicine (COLC), a tubulin-binding gout medication [26] caused a marked decrease in cell count at 30 nM, while only a slight decrease in ATP content was observed, also denoted by the calculated IC_50_ values (S8 Table). The decrease in cell count with COLC at 30 nM and higher was associated with a striking phenotypic change, as pictured in Fig 1. In these conditions, the formation of round multi-nucleated cells that do not elongate like myotubes was observed, which could indicate a potential stress response from the cells. Another example of perturbation with decreased cell count and no effect on ATP content was observed with FIAL in the three highest concentrations. These findings suggest that relying solely on ATP content might not fully capture the extent of cytotoxic effects, underscoring the importance of considering multiple assays to accurately assess cell health.

### Cell painting phenotypic features can predict cell count

#### Individual models to predict cell count.

**Fig 3:**
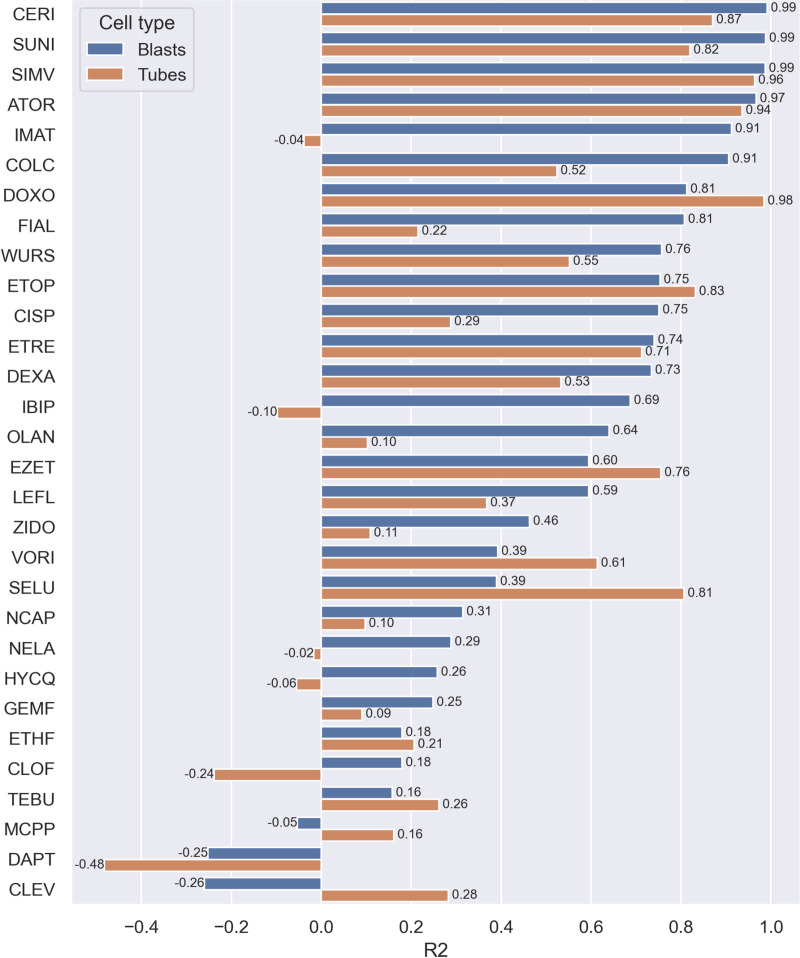
Prediction performance of cell count prediction models built on C2C12 myoblasts and myotubes on Harmony data. Test set determination coefficients (R2) of predicted cell counts against observed cell counts, from Random Forest models built for 30 compounds of the Toxifate library of suspected myotoxicants.

We next assessed how well the phenotypic profiles extracted with the CPA reflected changes in cell count and cell viability. Starting with the cell count data, a performance summary of 30 RF regression models built with each compound individually is given in Fig 3. The resulting models predicted the median cell count with determination coefficients R^2^ ranging from 0.269 (MCPP), up to 0.996 (CERI), with higher values meaning more accurate predictions. We observed a clear trend in the repartition of determination coefficients, as better performing models were built on compounds showing the most cytotoxic phenotypes, and hence the biggest drop in cell counts with comparison to non-toxic treatments (or controls). In a similar manner, the 5 worst performing models (DAPT, NCAP, EZET, CLEV, MCPP) were built on compounds showing no significant change in cell count in all tested concentrations. However, such high determination coefficients for all compounds could hide model overfitting, as many values above 0.5 would suggest an important statistical significance of predictions.

### Cell painting phenotypic features can predict ATP content/ cell viability

#### Individual models to predict ATP data.

**Fig 4:**
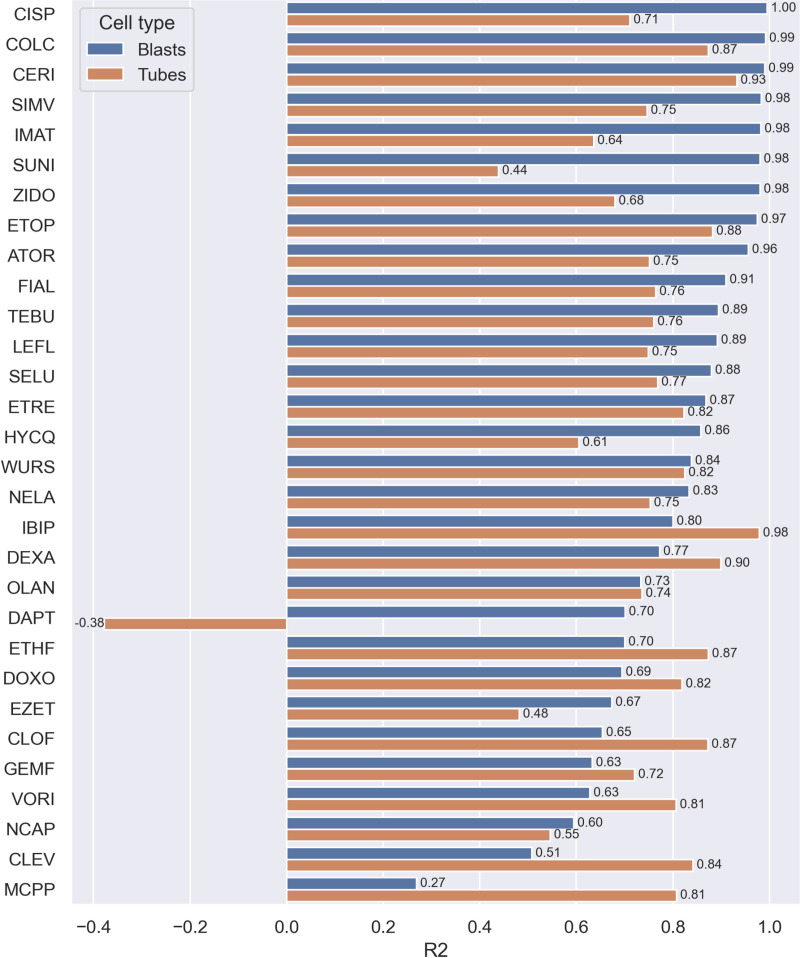
Prediction performance of cell viability prediction models built on C2C12 myoblasts and myotubes on Harmony data. Test set determination coefficients (R2) of predicted cell viability against observed viability, from Random Forest models built for 30 compounds of the Toxifate library. Luminescence data scaled against median luminescence of healthy DMSO controls (represented by 100% luminescence).

Next, we built models predicting cell viability deduced from the Cell Titer-Glo assay. A set of 30 RF models were built on individual compounds using the same approach as described in the cell count prediction methodology. The performance of models followed a trend comparable to the observations in the prediction of cell count (Fig 4), with best-performing models found in the drugs with the most cytotoxic potential, such as CERI, SUNI, and COLC.

**Fig 5:**
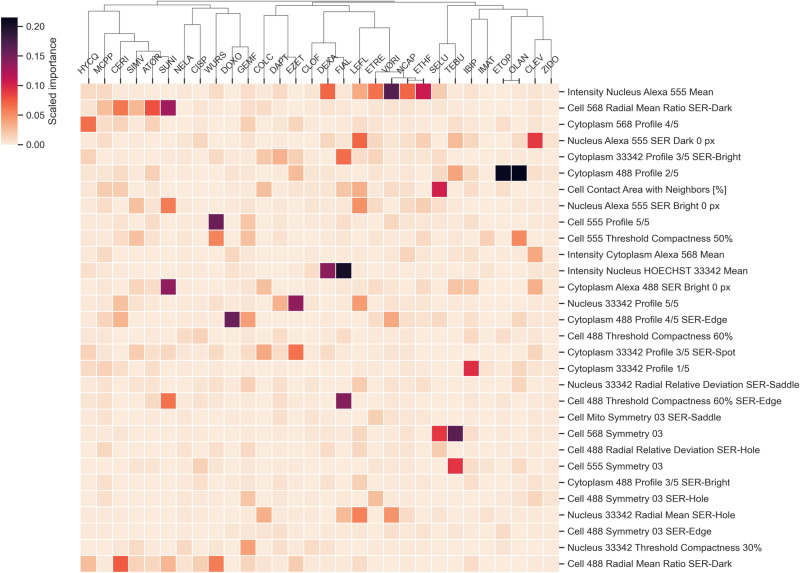
Heatmap and hierarchical clustering of top 30 Random Forest features importance for each model built on individual drugs for viability prediction in myoblasts. Features are ranked in descending order by their median rank of importance across all models (most important features are at the top of the heatmap). Clustering is performed using single-linkage and correlation metric. The lower half of the heatmap containing less prominent features was cropped for readability.

An in-depth analysis of the entire space of feature importance for each individual model showed that models of similar compounds yielded similar RF importances, with for instance SUNI and IMAT which were found to be closely clustered, as pictured in S5 Fig. ATOR, SIMV and CERI, the three HMGCR inhibitors were also found to populate the same subcluster, in a reduced set of feature importances, as seen in Fig 5. Remarkably, two subclusters in this figure highlighted the striking importance of only one feature each. The first subcluster of interest, composed by the compounds SIMV, ATOR, CERI and SUNI is marked by the high importance of the *Cell 568 Radial Mean Ratio SER-Dark* feature, one of the many texture properties extracted in the Harmony pipeline. This similarity between these drug models may indicate that similar phenotypic changes are induced with increasing concentrations of these four drugs. In a second subcluster of interest populated by ETRE, VORI, NCAP and ETHF, the feature *Intensity Nucleus Alexa 555 Mean* displays a far greater importance than for other models.

### RF model trained with all compounds

A single RF model was then trained on the entire drug panel on myoblast data, using well-level phenotypic profiles. The dataset is split into a training and a test set (80-20%), and yields a test set R^2^ of 0.861, and a mean squared error of 59.8, indicating that this model can predict cell viability with good accuracy (training set: R^2^ =  0.983, MSE =  6.67). The 10 most important features (according to mean decrease in impurity) of the RF model trained on Harmony data are shown in Table 1. An assessment of feature importance in the resulting RF shows that cytoplasm-related features are prevalent for cytotoxicity prediction, especially for Spots Edges and Ridges (SER) Profiles in the ER and mitochondria channels (“Cytoplasm Mito Profile 4/5 SER-Bright” and “Cytoplasm 488 Profile 4/5 SER-Edge”). In several models the feature describing the Nucleus profile in the Hoechst (DNA) channel (“Nucleus 33342 Profile 5/5”) was also crucial. A more extensive list of features and their importance is given in S7 Table.

**Table 1:** Summary of RF feature importance for the top 10 properties of the Random Forest model for viability prediction built on C2C12 myoblasts with the Harmony dataset.

Feature	RF Importance
Cytoplasm 488 Profile 4/5 SER-Edge	0.4474
Nucleus 33342 Profile 5/5	0.1604
Cytoplasm Mito Profile 4/5 SER-Bright	0.0829
Cell 488 Radial Mean Ratio SER-Dark	0.0264
Nucleus 33342 Radial Mean SER-Hole	0.0263
Cell Contact Area with Neighbors [%]	0.0087
Nucleus 33342 Axial Small Length SER-Hole	0.0080
Intensity Cytoplasm Alexa 568 Mean	0.0072
Cytoplasm 33342 Profile 3/5 SER-Saddle	0.0064
Cytoplasm 488 Profile 3/5 SER-Saddle	0.0056

#### Prediction of viability and cell count in myotubes.

The same approach was followed for predicting cell viability and cell count in C2C12 differentiated myotubes as seen in Fig 3 and 4, using the same segmentation algorithm in Harmony 4.8 as for myoblasts, but a different segmentation in the dedicated Cell Profiler pipeline. Model performance followed the same trends as the myoblasts, with better predictions given for most cytotoxic compounds, and less reliable models built on compounds displaying no significant signs of toxicity, as seen in Fig 2, hence these models are mainly capturing noise in the cell viability and cell count. No discrepancy in descriptor importance was noted, as the same feature classes were found with comparable importance.

#### Generation of new data points with SMOGN.

It can easily be pointed out that the dataset used in this study is quite unbalanced, as many treatments have little to no statistically significant effect on cell viability or cell count, leading to an underrepresentation of cytotoxic morphological profiles. To try overcoming this problem, a synthetic data generation algorithm such as SMOGN was used, to compensate for the unbalanced distribution of viabilities [27]. This algorithm is the generalization to regression tasks of the well-known SMOTE generative method, dedicated for classification models [28]. Promising results were obtained using the Python implementation, with the generation of new cytotoxic profiles with Gaussian noise applied to morphological features, and new balanced viability values are represented in S4 Fig. A global RF model trained on SMOGN augmented data reached a training R2 of 0.990, and a test R2 of 0.978, indicating an overwhelming prediction performance. The RF feature importance of this newly generated model was extracted, and displayed in S6 Table. Interestingly, these feature importances are very closely related to those of the original model, which validates the robustness of the synthetic phenotypes.

#### Cell Painting phenotypes uncovered unsuspected similarities between the drugs.

**Fig 6:**
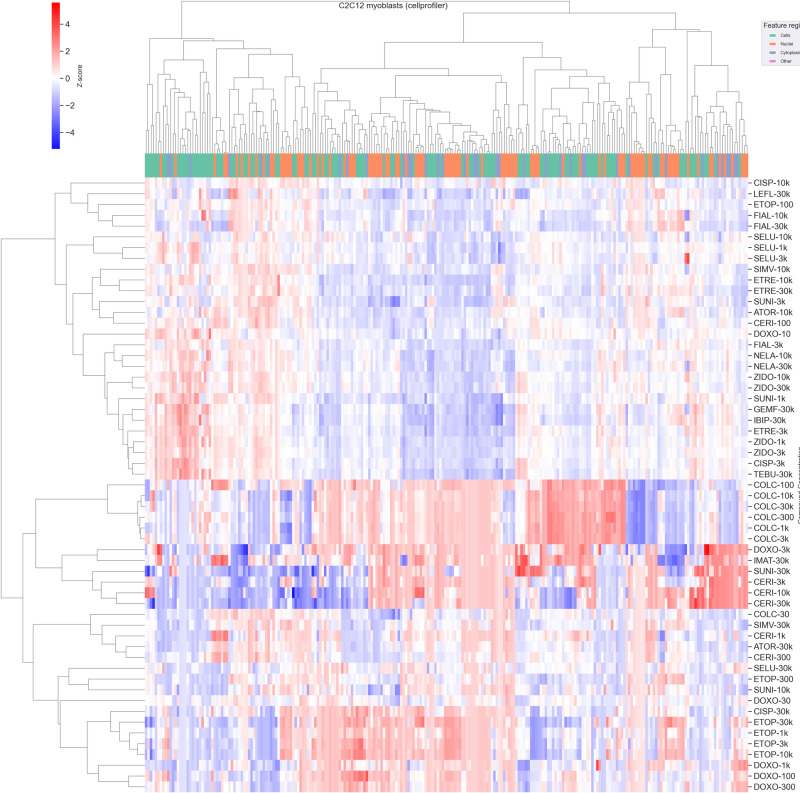
Heatmap and hierarchical clustering of treatment-level morphological profiles for C2C12 myoblasts. Only profiles with induction >  0.2 are represented. Hierarchical clustering of treatments and features computed with average-linkage on Pearson correlations. Z-scores clipped to ± 8 MAD for increased readability and color scaling.

To clearly represent the information encoded in each quantitative phenotype, and to identify relationships between different myotoxicants, a clustering of the normalized profiles was conducted. The results of this analysis are reported in Fig 6. Several observations can be made from the clustering of treatment conditions. Firstly, it can be pointed out that the two largest clusters obtained with this analysis are separating the condition phenotypes according to the magnitude of the change compared to the vehicle control: indeed, the top cluster only contains phenotypes with low deviations from the vehicle values, while the bottom cluster, which contains several cytotoxic conditions, displays larger variations from the control, as denoted by the deeper colors. Interestingly, a subcluster solely made of COLC conditions can be found in the middle part of the clustergram, which denotes the peculiar nature of the phenotypic changes induced by this gout medication. It is important to note that only major phenotypic perturbations were used for the clustering: the myoblast phenotypes that displayed an induction below 0.2 were ignored for the analysis. A profile induction of 0.2 is interpreted such as at least 20% of features in that phenotype are significantly affected. This induction threshold (20% affected features or greater) adapted from previous studies used for filtering out low-cytotoxic perturbations [29] was found to be efficient on our C2C12 data and excluded profiles with minor morphological changes in myoblasts and myotubes, that could have spoiled the clustering with meaningless clusters. The same analysis was also conducted without the induction filter as comparison, and is pictured in S6 Fig. From these observations, this threshold filter can be considered as a good balance between the removal of noisy profiles white maintaining strong signal from more distinct profiles.

The distribution of induction values for the two cell types is given in S3 Fig. The important number of dimensions in this dataset means only a few distance metrics are suitable for comparing the different phenotypes (each represented as a vector of decimal values). In this study, hierarchical clustering using the average linkage method and the Pearson correlation metric, yielded distinct clusters of toxic phenotypes. Euclidean distance was also tested, but this distance metric was excluded due to the “curse of dimensionality” [30] arising with such high dimensionality. The clustergram obtained with Euclidean distance is displayed in S7 Fig. Mahalanobis distance was also considered, but its usage was limited by the total number of features being greater than the number of samples (which is frequent in cell phenotype analysis), making its use impossible.

Several expected clusters emerged from the analysis. For instance, statins grouped together even though their chemical structures are dissimilar and do not follow a clear scaffold, which is consistent with the effects being mediated through their common molecular target (3-hydroxy-3-methylglutaryl-CoA reductase;HMGCR). More surprisingly, SUNI, a tyrosine kinase inhibitor (TKi) which has no structural similarity to statins and has a different pharmacological target. also clustered with the statins.

Previous studies have shown the cytotoxic potential of SUNI in C2C12 cells [31]. Several cases of SUNI induced rhabdomyolysis have been reported in the past [32], which is one of the most serious adverse effects observed globally with statins, and many lipid-lowering drugs. To further investigate the relationship between SUNI and the statin family, a connectivity search on gene expression data was conducted on cMAP [33], accessed from the clue.io platform. The results of this connectivity analysis are reported in S2 Table. A comparison of SUNI with the three statins from our panel displayed many similarities on the perturbagen class and on targets. It was also noted in this cMAP analysis, that SUNI and statins also seem to share similarities in gene expression with dasatinib, a proto-oncogene tyrosine-protein kinase (SRC) inhibitor, and a norepinephrine reuptake inhibitor. The genetic connectivity was less conclusive, with only a few genes of interest targeted by the four compounds, like PCSK9 (Proprotein convertase subtilisin/kexin type 9) a target for the treatment of dyslipidemia, or MED4 (mediator of RNA polymerase II transcription subunit 4, also known as DRIP36). From these different sources as well as the present study, one could suggest that SUNI shares toxicological similarities with HMGCR inhibitors.

## Discussion

CPA identifies chemical hazards causing hepatotoxicity [34], nephrotoxicity [35] and neurotoxicity [36]. In some cases CPA approaches use cell lines relevant to the target organ of interest (e.g.,), or in other cases a cell line suited to CPA, but unrelated to the target organ of interest are used. As organ specific gene expression is often a key determinant of target organ toxicity, CPA that employ cell lines relevant to the target organ are expected to be a better in vitro model. Therefore, CPA to identify myotoxicants should employ a relevant cell model, like C2C12. This is the first CPA using myotoxicants and muscle-relevant cells, and it identifies both strengths and limitations of the approach, while providing a foundation for future improvements of in vitro prediction of myotoxicity.

Here, 30 myotoxicants (out of approximately 200 known myotoxicants [37]) were tested at a range of concentrations to assess whether CPA 1: detected toxicant induced changes in a muscle-relevant cell line and 2: the concentration dependence of any effect and its relationship to reported points of departure. As a proof-of-principle, the ability of CPA to predict cytotoxicity measured by a commonly used cell viability assay was assessed. Although 30 compounds is a small sample (other CPA toxicity studies assess up to 30 000 chemicals [10]), the chemicals were chosen to represent diverse structures, molecular targets and mechanisms of toxicity. Also, they were tested at a range of concentrations, where other studies typically chose a single concentration.

This allowed detection of concentration dependent differences in phenotypes. For instance, Imatinib followed a trend of morphological perturbations similar to simvastatin in most concentrations but appeared very different at 300 nM, and then even got close to a high dose cerivastatin-like phenotype at 30 µ M. Our findings highlight the challenge of choosing the “right” concentration for testing a single dose faced by other CPA studies. A common approach in these studies is to use the LOAEL or NOAEL (lowest or no observed adverse effect level) for each compound, but this presupposes that the point of departure values are known and could miss valuable information on potentially unsuspected pathways activated at specific concentrations.

In several cases, major discrepancies between the cell viability of a treatment and the corresponding cell count were seen. For instance, a treatment of C2C12 myoblasts with cisplatin seems to have an important impact on the number of cells detected in a treatment well, whereas ATP content of the cell population was not significantly affected, as seen in Fig 2. Cisplatin’s effect on the cell cycle, which is the basis of its anti-tumour activity, probably explains these observations. The implication is that CISP could inhibit muscle regeneration by preventing the proliferation of satellite cells. Various mechanisms are proposed for the cisplatin-induced muscle wasting and body weight loss in cancer patients [38], but an explanation involving satellite cell proliferation means that separating cisplatin’s antitumor effect from its myotoxicity will be very challenging. This would imply that approaches to prevent cisplatin-induced cachexia by increasing appetite and decreasing catabolism are unlikely to work.

In another example, COLC caused a major phenotypic change associated with the appearance of multinucleated myoblasts without a marked change in cellular ATP levels. These cells are similar to multinucleated cells formed by differentiating muscle cells from chick embryo breast muscle that were treated with 10 nM COLC [39]. Previous studies have shown the cytotoxic potential of SUNI in C2C12 cells [31] and several cases of SUNI induced rhabdomyolysis have been reported [32]. The phenotypic similarity of SUNI to the statins, supported by pathway analysis, suggests that these effects of SUNI are mediated by a hitherto unsuspected effect on HMGCR.

NELA is commonly used as a treatment for T-cell acute lymphoblastic leukemia, while ZIDO is used as an HIV treatment and preventive medication, targeting the HIV-reverse transcriptase (38). Both compounds are nucleoside analogues and share the inhibition of DNA synthesis as a common mechanism of action. ZIDO is a thymidine analogue, with an azide substituent replacing the cyclic alcohol group. NELA is a purine nucleoside analogue with an additional OH group added to the sugar ring, and a methoxy group replacing the cyclic ketone.

## Limitations

The implementation of this high-content microscopy assay raised important challenges on diverse aspects of the project, from microscopy to computational modeling. As seen in this study, such tasks of building predictive models for toxicity endpoints can be hindered by class imbalance, or imbalance of value distribution in the regression tasks. Data generation algorithms such as SMOTE or SMOGN can be used to alleviate this problem, but these newly generated data points cannot replace true biological data. The ideal solution could be to modify the experimental approach, to account for more cytotoxic profiles, for instance by including compounds with fewer reports linked to toxic myopathies, and also avoiding compounds with no visible effect on cell morphology, or no effect on viability.

It is to be noted that the choice of the right model of microscopy plate is of utmost importance for this kind of assay, especially when cell adherence is a crucial factor, as we noticed that myotube shape was different between plate models. Slight differences in plate specification, manufacturer, or even manufacturing batch can lead to significant phenotypic differences. This phenomenon was mainly observed when capturing myotube data (as their shape is very dependent on the well coating). Mitigation is possible with rigorous normalization, including techniques such as Robust MAD, and B-score plate normalization [34].

Further studies should pinpoint specific features or features groups in the morphological phenotypes to correlate precise changes in morphology and stress signaling pathways or early signs of cytotoxicity, as already introduced by a few studies to date [40]. This step will allow us to capture all the necessary information to potentially describe and predict differential gene expression, or more broadly pathway activation and inhibition.

To conclude, we report that this study is, to our knowledge, the first application of the CPA to the C2C12 cell line. By studying myoblasts and myotubes, it provides information of two distinct muscle cell types and a foundation for the development of improved in vitro assays for myotoxicity.

## Materials and methods

### Drug library

The search terms “myotoxicity” and “rhabdomyolysis” combined with “drug” or “toxicant” were used to identify the relevant literature. Toxicants reported in this literature were then investigated for the reported mechanism of action to identify the molecular target/molecular initiating event. Toxicants that targeted the neuromuscular junction or the nervous system were excluded. Toxicants reported to have a direct action on muscle cells, or where the target was unclear were included. A final collection of 30 myotoxicants (Table 2) was prepared in a 96-well plate format in five motherplates, each containing six compounds. The toxicants were dissolved in DMSO (or DMF for cisplatin) at a range of 8 concentrations from 10 µ M to 30 mM and three replica plates were prepared. The plates were sealed and stored at -20 °C until use. Plates were thawed at room temperature in the dark for at least 2 hours before use.

**Table 2:** Toxifate drug panel of myotoxicants.

Drug name	Class	Abbreviation	Supplier	Chembl ID
Aminocaproic acid	Anti-fibrinolytic	**NCAP**	Sigma	CHEMBL1046
Atorvastatin	Lipid lowering	**ATOR**	Sigma	CHEMBL1487
Cerivastatin	Lipid lowering	**CERI**	Sigma	CHEMBL1477
Cisplatin	Chemotherapy	**CISP**	Sigma	CHEMBL11359
Clevudine	Hepatitis B	**CLEV**	Santa Cruz Biotech	CHEMBL458875
Clofibric acid	Lipid lowering	**CLOF**	Sigma	CHEMBL683
Colchicine	Gout	**COLC**	Sigma	CHEMBL107
Daptomycin	Antibiotic	**DAPT**	Sigma	CHEMBL387675
Dexamethasone	Corticosteroid	**DEXA**	Sigma	CHEMBL384467
Doxorubicin	Chemotherapy	**DOXO**	Cayman Chemicals	CHEMBL53463
Ethyl fluoroacetate	Rodenticide	**ETHF**	Sigma	CHEMBL509273
Etoposide	Chemotherapy	**ETOP**	Sigma	CHEMBL44657
Etretinate	Retinoid	**ETRE**	Sigma	CHEMBL464
Ezetimibe	Lipid lowering	**EZET**	Sigma	CHEMBL1138
Fialuridine	Hepatitis B	**FIAL**	Sigma	CHEMBL271475
Gemfibrozil	Lipid lowering	**GEMF**	Sigma	CHEMBL457
Hydroxychloroquine	Anti-malarial	**HYCQ**	Sigma	CHEMBL1535
Ibipinabant	Cannabinoid	**IBIP**	Santa Cruz Biotech	CHEMBL412262
Imatinib	Chemotherapy	**IMAT**	Sigma	CHEMBL941
Leflunomide	Anti-rheumatic	**LEFL**	Sigma	CHEMBL960
Mecoprop	Herbicide	**MCPP**	Sigma	CHEMBL272942
Nelarabine	Chemotherapy	**NELA**	Sigma	CHEMBL1201112
Olanzapine	Antipsychotic	**OLAN**	Sigma	CHEMBL715
Selumetinib	Chemotherapy	**SELU**	Santa Cruz Biotech	CHEMBL1614701
Simvastatin	Lipid lowering	**SIMV**	Cayman Chemicals	CHEMBL1064
Sunitinib	Chemotherapy	**SUNI**	Cayman Chemicals	CHEMBL535
Tebuconazole	Antifungal	**TEBU**	Sigma	CHEMBL487186
Voriconazole	Antifungal	**VORI**	Sigma	CHEMBL638
Wurster’s blue	Redox indicator	**WURS**	Sigma	CHEMBL1393325
Zidovudine	Antiretroviral	**ZIDO**	Sigma	CHEMBL129

### C2C12 cell culture+

Proliferating C2C12 myoblasts (Sigma-Aldrich, CRL-1772) were cultured in a solution of Dulbecco’s Modified Eagle Medium (DMEM) (Sigma) supplemented with 20% Fetal Bovine Serum (FBS) (Sigma) and an antibiotic cocktail of 1% Penicillin and Streptomycin (PS) (Sigma Aldrich). Cells were passaged at 60-70% confluence. Cells to be differentiated into myotubes were seeded at 5,000 cells per well into 384 wells plates (Greiner Bio-One µ Clear) using a BioTek Multiflo automated dispenser at 20 µ L per well. Cells were cultured for 4 days in a differentiation medium (DM; DMEM supplemented with 2% Horse Serum (Sigma) and 1% PS) before being treated with drugs. Myoblasts were seeded at 1,000 cells per well on day 4 in a growth medium (GM; DMEM supplemented with 20% Fetal Bovine Serum and 1% PS).

### Drug treatment

The two C2C12 cell types were treated on day 4 using a PerkinElmer JANUS automation system for optimal experimental reproducibility. Cells were treated with toxicants at 8 different concentrations from 10 nM to 30 µ M. The final concentration of the vehicle (DMSO for all drugs except cisplatin, which was dissolved in DMF) was 0.1%. Plates were then incubated at 37°C and 5% CO_2_ for 72 hours in the dark. Three biological replicates run on separate days were completed with 4 technical replicates of each condition on each plate, over 5 well sites. Each perturbation (one drug at one concentration) was hence repeated in 12 wells to capture a large population of cell phenotypes.

### Cell Painting staining and image acquisition

**Fig 7:**
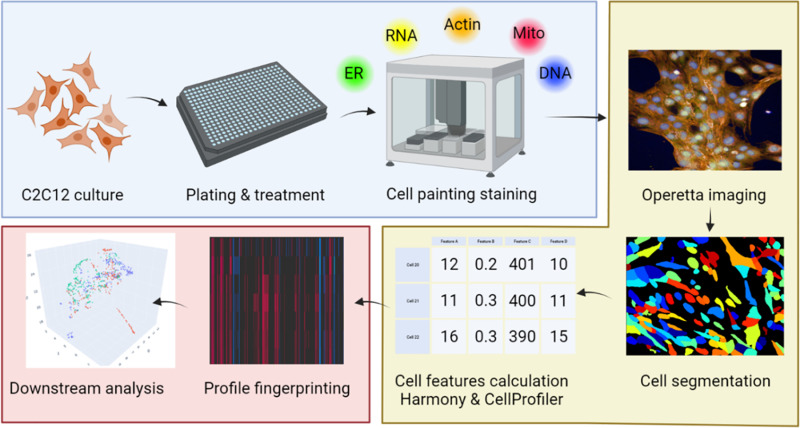
Outline of the CPA protocol applied to C2C12 cells. Color coding, Blue: cell culture and staining, Yellow: high-content microscopy acquisition, Red: data analysis.

PhenoVue Cell Painting kits (ref. PING22) were obtained from PerkinElmer (MA, USA; now Revvity). At 72 hours, the cells were fixed and stained following the JUMP CPA protocol from Carpenter lab (Fig 7), using the JANUS automated dispensing platform. The list of reagents used for this assay and final concentrations can be found in S3 Table. Additionally, the full Cell Painting staining protocol is detailed in S4 Table. After staining, the plates were then imaged using a Perkin Elmer Operetta high content microscope, equipped with a 20 ×  long air objective. Five fields were captured per well, with no pixel binning. Images were analyzed with Harmony 4.8 following the building blocks script described in Nyffeler et al. [24], and then analyzed externally using the open-source Cell Profiler software version 4.2.7 [41] using in-house pipelines. A summary of this protocol is outlined in Fig 7. The illumination correction and analysis Cell Profiler pipelines can be found on this project GitHub repository.

### Data analysis

Data analysis was performed in Python 3.10 with the pycytominer package for per-well profile aggregation and normalization [42,43]. Normalization methods used were min-max scaling, Z-score, and Robust MAD, as implemented in pycytominer. The well-level phenotypes were then aggregated from the single-cell data, by taking the median feature values for each well population, and then normalizing on the negative vehicle control wells (30 to 40 wells per plate) with a Z-score method. Cell counts per well were also calculated for comparison with the cell viability assay. Statistical significance of cell count and cell viability for each compound and concentration was assessed by performing an independent T-test against the vehicle controls, and a p value threshold of 0.05 was retained.

### Profile clustering

Perturbation-aggregated profiles were clustered using hierarchical clustering, with complete linkage method. The value of induction of a well-level profile was computed as the percentage of morphological features with an absolute Z-score value greater than 3, as introduced by Schneidewind et al [29]:


$$Induction\left(\%\right)=\frac{Nfeatureswith\left|Zscore\right|>3}{Totalnumberoffeatures}*100.$$

This metric allows a simple filtering of perturbations with low phenotypical significance and high noise.

### Cell viability assay

The same procedure for cell culture and drug treatment as the CPA was followed for assessing cellular viability with ATP content. After 72 h of treatment, 20 µ L of Cell Titer-Glo reagent (Promega, Walldorf, Germany) was added to samples, plates were incubated in the dark at room temperature for 30 minutes, and luminescence was read with a Perkin Elmer VICTOR3 plate reader. Data was exported as an XLS file containing raw luminescence reads, which was then normalized, and expressed as a percentage of viability of each plate vehicle controls (DMSO).

### Viability prediction model building

The median cell count per well was calculated with the number of valid objects segmented during image analysis, processed with a custom imaging pipeline set up in Harmony. Phenotypes pre-processing and model building are conducted in Python 3.10 and scikit-learn [44]. Features presenting standard deviation higher than 2,000 were discarded, to remove potentially large values caused by the normalization process. Correlated features were also removed with a threshold of 0.9. An individual RF regression model [45] was built for each toxicant (excluding DMSO controls) using all remaining features, and each model was validated by a test set using random sampling of well-level phenotypes (train test ratio of 80/20). 96 observations were hence studied for each toxicant (8 concentrations ×  4 wells per plate ×  3 plate replicates). The scikit-learn algorithm implementation of Random Forests was used for model building [44]. Each RF model was trained using 200 estimators (trees), bootstrapping was turned on, and no limit to maximum depth was set. A random seed was used (42) to allow for replicability of this experiment. All other parameters were kept as default. Data generation was performed via with the SMOGN algorithm in the dedicated section, and the parameters were set to the following: rel_xtrm_type =  “low”, samp_method = ‘balance’, rel_coef =  5, and rel_thres =  0.8

## Supporting information

S1 Table Summary of extracted features with Harmony and CellProfiler software. (A) Summary of features extracted via the Harmony analysis building blocks, following guidelines from Nyffeler et al. Cell positional features and basic morphology were computed and omitted from this table for readability. (B) Summary of features extracted with CellProfiler. Cells are segmented into 5 compartments in Harmony, and in 3 with CellProfiler (nuclei, cytoplasm, and cell). Nomenclature of cell regions: N =  nuclei; Ce =  cell; Cy =  cytoplasm; M =  membrane; R =  ring region, x = all compartments.
(PDF)

S2 Table Connectivity results of sunitinib against the statins ATOR, CERI, SIMV obtained from the cMAP tool ([clue.io]). cMAP tau scores of top perturbagen classes (A), top genes (B), and top compound similarities (C). Scores ranging from -100 to + 100, and values over 90 or below -90 are considered of interest for further investigation. Values outside this threshold are outlined in red. Only compound BRD-M64432851 was considered for sunitinib due to the presence of duplicates. Duplicate compound names were truncated for readability.
(PDF)

S3 Table List of reagents used for conducting the Cell Painting Assay.
(PDF)

S4 Table Cell Painting Protocol for C2C12 cells, adapted from the standard Cell Painting protocol (v3). (PDF)

S5 Table Summary of the best RF feature importances extracted from the global RF model generated on myoblast Harmony dataset, with SMOGN data augmentation. (PDF)

S6 Table Top RF importance of the global RF model. Built on C2C12 Harmony myoblast data, with no data augmentation
(PDF)

S7 Table Summary of IC50 and LC50 values. Obtained with the cell count analysis from Harmony data, and from the CellTiter-Glo cell viability assay.
(PDF)

S1 Fig Analysis of C2C12 nuclei area following treatments with colchicine (A) Concentration dependent changes in myoblast nuclear area induced by Colchine (COLC) compared the effects of a vehicle control (DMSO). Aminocaproic acid (NCAP) is a control drug. (B) Concentration dependent changes in myoblast and myotube nuclear area induced by Etoposide (ETOP). The figure can be reproduced from 220412 and 220425 data sets using the accompanying code in S1 Appendix.
(PNG)

S2 Fig Results of a document search on the Clarivate Web of Science platform. Analysis performed on August 1st 2023. Results given in percentage of total documents yielded with all queries. Keywords were entered as displayed in lowercase, and no synonyms were considered. Search conducted in the WoS Core Collection only, including all document types.
(PNG)

S3 Fig Distribution of induction values for myoblasts (left) and myotubes (right) treatment-level data. (PNG)

S4 Fig Density plot of the distribution of viability values for C2C12 myoblasts. Treatment-level data, before data augmentation (blue, Original) and after data augmentation using the SMOGN algorithm (orange, SMOGN).
(PNG)

S5 Fig Heatmap and hierarchical clustering of all remaining Random Forest features importance for each model built on individual drugs for viability prediction in myoblasts. Features are ranked in descending order by their median rank of importance across all models (most important features are at the top of the heatmap). Clustering is performed using single-linkage and correlation metric. The lower half of the heatmap containing less prominent features was cropped for readability.
(PNG)

S6 Fig Heatmap and hierarchical clustering of treatment-level morphological profiles for C2C12 myoblasts. All profiles are represented, no induction filter. Hierarchical clustering of treatments and features computed with average-linkage on Pearson correlations. Z-scores clipped to ± 8 MAD for increased readability and color scaling.
(PNG)

S7 Fig Heatmap and hierarchical clustering of treatment-level morphological profiles for C2C12 myoblasts. Profiles with induction >  0.2 are represented. Hierarchical clustering of treatments and features computed with average-linkage on Euclidean distances. Z-scores clipped to ± 8 MAD for increased readability and color scaling.
(PNG)

S1 Appendix Analysis of Colchicine data. (PDF)

## References

[1] Janssen L, Allard NAE, Saris CGJ, Keijer J, Hopman MTE, Timmers S. Muscle toxicity of drugs: when drugs turn physiology into pathophysiology. Physiol Rev. 2020;100(2):633–72. doi: 10.1152/physrev.00002.2019 31751166

[2] Jones JD, Kirsch HL, Wortmann RL, Pillinger MH. The causes of drug-induced muscle toxicity. Curr Opin Rheumatol. 2014;26(6):697–703. doi: 10.1097/BOR.0000000000000108 25191992

[3] Pognan F, Beilmann M, Boonen HCM, Czich A, Dear G, Hewitt P, et al. The evolving role of investigative toxicology in the pharmaceutical industry. Nat Rev Drug Discov. 2023;22(4):317–35. doi: 10.1038/s41573-022-00633-x 36781957 PMC9924869

[4] O’Brien PJ, Irwin W, Diaz D, Howard-Cofield E, Krejsa CM, Slaughter MR, et al. High concordance of drug-induced human hepatotoxicity with in vitro cytotoxicity measured in a novel cell-based model using high content screening. Arch Toxicol. 2006;80(9):580–604. doi: 10.1007/s00204-006-0091-3 16598496

[5] Furberg CD, Pitt B. Withdrawal of cerivastatin from the world market. Curr Control Trials Cardiovasc Med. 2001;2(5):205–7. doi: 10.1186/cvm-2-5-205 11806796 PMC59524

[6] Law M, Rudnicka AR. Statin safety: a systematic review. Am J Cardiol. 2006;97(8A):52C-60C. doi: 10.1016/j.amjcard.2005.12.010 16581329

[7] Jang Y-O, Quan X, Das R, Xu S, Chung C-H, Ahn CM, et al. High-dose clevudine impairs mitochondrial function and glucose-stimulated insulin secretion in INS-1E cells. BMC Gastroenterol. 2012;12:4. doi: 10.1186/1471-230X-12-4 22230186 PMC3288815

[8] Carr A, Cooper DA. Adverse effects of antiretroviral therapy. Lancet. 2000;356(9239):1423–30. doi: 10.1016/S0140-6736(00)02854-3 11052597

[9] Higuchi I, Izumo S, Kuriyama M, Suehara M, Nakagawa M, Fukunaga H, et al. Germanium myopathy: clinical and experimental pathological studies. Acta Neuropathol. 1989;79(3):300–4. doi: 10.1007/BF00294665 2609936

[10] Bray M-A, Singh S, Han H, Davis CT, Borgeson B, Hartland C, et al. Cell Painting, a high-content image-based assay for morphological profiling using multiplexed fluorescent dyes. Nat Protoc. 2016;11(9):1757–74. doi: 10.1038/nprot.2016.105 27560178 PMC5223290

[11] Akbarzadeh M, Deipenwisch I, Schoelermann B, Pahl A, Sievers S, Ziegler S, et al. Morphological profiling by means of the Cell Painting assay enables identification of tubulin-targeting compounds. Cell Chem Biol. 2022;29(6):1053-1064.e3. doi: 10.1016/j.chembiol.2021.12.009 34968420

[12] Lin S, Liu H, Svenningsen EB, Wollesen M, Jacobsen KM, Andersen FD, et al. Expanding the antibacterial selectivity of polyether ionophore antibiotics through diversity-focused semisynthesis. Nat Chem. 2021;13(1):47–55. doi: 10.1038/s41557-020-00601-1 33353970 PMC7610524

[13] Bray M-A, Gustafsdottir SM, Rohban MH, Singh S, Ljosa V, Sokolnicki KL, et al. A dataset of images and morphological profiles of 30 000 small-molecule treatments using the Cell Painting assay. Gigascience. 2017;6(12):1–5. doi: 10.1093/gigascience/giw014 28327978 PMC5721342

[14] Salucci S, Battistelli M, Burattini S, Squillace C, Canonico B, Gobbi P, et al. C2C12 myoblast sensitivity to different apoptotic chemical triggers. Micron. 2010;41(8):966–73. doi: 10.1016/j.micron.2010.07.002 20674376

[15] Bonifacio A, Sanvee GM, Brecht K, Kratschmar DV, Odermatt A, Bouitbir J, et al. IGF-1 prevents simvastatin-induced myotoxicity in C2C12 myotubes. Arch Toxicol. 2017;91(5):2223–34. doi: 10.1007/s00204-016-1871-z 27734117

[16] Bouitbir J, Panajatovic MV, Frechard T, Roos NJ, Krähenbühl S. Imatinib and dasatinib provoke mitochondrial dysfunction leading to oxidative stress in C2C12 myotubes and human RD Cells. Front Pharmacol. 2020;11:1106. doi: 10.3389/fphar.2020.01106 32792947 PMC7390871

[17] Willkomm L, Schubert S, Jung R, Elsen M, Borde J, Gehlert S, et al. Lactate regulates myogenesis in C2C12 myoblasts in vitro. Stem Cell Res. 2014;12(3):742–53. doi: 10.1016/j.scr.2014.03.004 24735950

[18] Schöneich C, Dremina E, Galeva N, Sharov V. Apoptosis in differentiating C2C12 muscle cells selectively targets Bcl-2-deficient myotubes. Apoptosis. 2014;19(1):42–57. doi: 10.1007/s10495-013-0922-7 24129924 PMC3939833

[19] Murray TVA, McMahon JM, Howley BA, Stanley A, Ritter T, Mohr A, et al. A non-apoptotic role for caspase-9 in muscle differentiation. J Cell Sci. 2008;121(Pt 22):3786–93. doi: 10.1242/jcs.024547 18957517

[20] Hughes RE, Elliott RJR, Li X, Munro AF, Makda A, Carter RN, et al. Multiparametric high-content cell painting identifies copper ionophores as selective modulators of esophageal cancer phenotypes. ACS Chem Biol. 2022;17(7):1876–89. doi: 10.1021/acschembio.2c00301 35696676 PMC9295120

[21] Schorpp K, Bessadok A, Biibosunov A, Rothenaigner I, Strasser S, Peng T, et al. CellDeathPred: a deep learning framework for ferroptosis and apoptosis prediction based on cell painting. Cell Death Discov. 2023;9(1):277. doi: 10.1038/s41420-023-01559-y 37524741 PMC10390533

[22] Nyffeler J, Willis C, Harris FR, Taylor LW, Judson R, Everett LJ, et al. Combining phenotypic profiling and targeted RNA-Seq reveals linkages between transcriptional perturbations and chemical effects on cell morphology: Retinoic acid as an example. Toxicol Appl Pharmacol. 2022;444:116032. doi: 10.1016/j.taap.2022.116032 35483669 PMC10894461

[23] Schölermann B, Bonowski J, Grigalunas M, Burhop A, Xie Y, Hoock JGF, et al. Identification of Dihydroorotate Dehydrogenase Inhibitors Using the Cell Painting Assay. Chembiochem. 2022;23(22):e202200475. doi: 10.1002/cbic.202200475 36134475 PMC9828254

[24] Nyffeler J, Willis C, Lougee R, Richard A, Paul-Friedman K, Harrill JA. Bioactivity screening of environmental chemicals using imaging-based high-throughput phenotypic profiling. Toxicol Appl Pharmacol. 2020;389:114876. doi: 10.1016/j.taap.2019.114876 31899216 PMC8409064

[25] Cree IA, Andreotti PE. Measurement of cytotoxicity by ATP-based luminescence assay in primary cell cultures and cell lines. Toxicol In Vitro. 1997;11(5):553–6. doi: 10.1016/s0887-2333(97)00060-x 20654351

[26] Finkelstein Y, Aks SE, Hutson JR, Juurlink DN, Nguyen P, Dubnov-Raz G, et al. Colchicine poisoning: the dark side of an ancient drug. Clin Toxicol (Phila). 2010;48(5):407–14. doi: 10.3109/15563650.2010.495348 20586571

[27] Branco P, Torgo L, Ribeiro RP. SMOGN: a pre-processing approach for imbalanced regression. proceedings of the first international workshop on learning with imbalanced domains: theory and applications. PMLR; 2017. p. 36–50. Available: https://proceedings.mlr.press/v74/branco17a.html

[28] Chawla NV, Bowyer KW, Hall LO, Kegelmeyer WP. SMOTE: Synthetic Minority Over-sampling Technique. jair. 2002;16:321–57. doi: 10.1613/jair.953

[29] Schneidewind T, Brause A, Pahl A, Burhop A, Mejuch T, Sievers S, et al. Morphological profiling identifies a common mode of action for small molecules with different targets. Chembiochem. 2020;21(22):3197–207. doi: 10.1002/cbic.202000381 32618075 PMC7754162

[30] Assent I. Clustering high dimensional data. WIREs Data Min & Knowl. 2012;2(4):340–50. doi: 10.1002/widm.1062

[31] Damaraju VL, Kuzma M, Cass CE, Putman CT, Sawyer MB. Multitargeted kinase inhibitors imatinib, sorafenib and sunitinib perturb energy metabolism and cause cytotoxicity to cultured C2C12 skeletal muscle derived myotubes. Biochem Pharmacol. 2018;155:162–71. doi: 10.1016/j.bcp.2018.07.001 29983397

[32] Liman AD, Passero VA, Liman AK, Shields J. A Rare case of sunitinib-induced rhabdomyolysis in renal cell carcinoma. Case Rep Oncol Med. 2018;2018:3808523. doi: 10.1155/2018/3808523 30123592 PMC6079559

[33] Subramanian A, Narayan R, Corsello SM, Peck DD, Natoli TE, Lu X, et al. A next generation connectivity map: L1000 platform and the first 1,000,000 profiles. Cell. 2017;171(6):1437–52.e17. doi: 10.1016/j.cell.2017.10.049 29195078 PMC5990023

[34] Lejal V, Cerisier N, Rouquié D, Taboureau O. Assessment of drug-induced liver injury through cell morphology and gene expression analysis. Chem Res Toxicol. 2023;36(9):1456–70. doi: 10.1021/acs.chemrestox.2c00381 37652439 PMC10523580

[35] Su R, Xiong S, Zink D, Loo L-H. High-throughput imaging-based nephrotoxicity prediction for xenobiotics with diverse chemical structures. Arch Toxicol. 2016;90(11):2793–808. doi: 10.1007/s00204-015-1638-y 26612367 PMC5065616

[36] Culbreth M, Nyffeler J, Willis C, Harrill JA. Optimization of human neural progenitor cells for an imaging-based high-throughput phenotypic profiling assay for developmental neurotoxicity screening. Front Toxicol. 2022;3:803987. doi: 10.3389/ftox.2021.803987 35295155 PMC8915842

[37] Wen Z, Liang Y, Hao Y, Delavan B, Huang R, Mikailov M, et al. Drug-Induced Rhabdomyolysis Atlas (DIRA) for idiosyncratic adverse drug reaction management. Drug Discov Today. 2019;24(1):9–15. doi: 10.1016/j.drudis.2018.06.006 29902520 PMC7050640

[38] Conte E, Bresciani E, Rizzi L, Cappellari O, De Luca A, Torsello A, et al. Cisplatin-induced skeletal muscle dysfunction: mechanisms and counteracting therapeutic strategies. Int J Mol Sci. 2020;21(4):1242. doi: 10.3390/ijms21041242 32069876 PMC7072891

[39] BischoffR, HoltzerH. The effect of mitotic inhibitors on myogenesis in vitro. J Cell Biol. 1968;36(1):111–27. doi: 10.1083/jcb.36.1.11119866713

[40] Seal S, Carreras-Puigvert J, Trapotsi M-A, Yang H, Spjuth O, Bender A. Integrating cell morphology with gene expression and chemical structure to aid mitochondrial toxicity detection. Commun Biol. 2022;5(1):858. doi: 10.1038/s42003-022-03763-5 35999457 PMC9399120

[41] Stirling DR, Swain-Bowden MJ, Lucas AM, Carpenter AE, Cimini BA, Goodman A. CellProfiler 4: improvements in speed, utility and usability. BMC Bioinformatics. 2021;22(1):433. doi: 10.1186/s12859-021-04344-9 34507520 PMC8431850

[42] Way G, Chandrasekaran SN, Bornholdt M, Fleming S, Tsang H, Adeboye A, et al. Pycytominer: Data processing functions for profiling perturbations. Available: https://github.com/cytomining/pycytominer

[43] Serrano E, Chandrasekaran S, Bunten D, Brewer K, Tomkinson J, Kern R, et al. Reproducible image-based profiling with Pycytominer. arXiv. 2024. 10.1038/s41592-025-02611-8 PMC12121495 40032995

[44] Pedregosa F, Varoquaux G, Gramfort A, Michel V, Thirion B, Grisel O. Scikit-learn: Machine learning in Python. J Mach Learn Res. 2011;12:2825–30.

[45] Breiman L. Random forests. Mach Learn. 2001;45(1):5–32. doi: 10.1023/a:1010933404324

