# Supplementary material for: Drug-induced cytotoxicity prediction in muscle cells, an application of the Cell Painting assay
Source: PLoS One. 2025 Mar 31;20(3):e0320040. doi: 10.1371/journal.pone.0320040 (PMC11957314; doi:10.1371/journal.pone.0320040)
Supplement: S1 Table — (A) Summary of features extracted via the Harmony analysis building blocks, following guidelines from Nyffeler et al. Cell positional features and basic morphology were computed and omitted from this table for readability. (B) Summary of features extracted with CellProfiler. Cells are segmented into 5 compartments in Harmony, and in 3 with CellProfiler (nuclei, cytoplasm, and cell). Nomenclature of cell regions: N = nuclei; Ce = cell; Cy = cytoplasm; M = membrane; R = ring region, x = all compartments. (PDF) [file pone.0320040.s001.pdf]

S1 Table: Summary of extracted features with Harmony and CellProfiler software. (A) Summary of features extracted via the Harmony analysis building blocks, following guidelines from Nyffeler et al. Cell positional features and basic morphology were computed and omitted from this table for readability. (B) Summary of features extracted with CellProfiler. Cells are segmented into 5 compartments in Harmony, and in 3 with CellProfiler (nuclei, cytoplasm, and cell). Nomenclature of cell regions: N = nuclei; Ce = cell; Cy = cytoplasm; M = membrane; R = ring region, x=all compartments.

(A)

Channel	Symmetry	Compactness	Axial	Radial	Profile	Intensity	Texture
DNA	N	N	N	N Ce	N Cy	N	N
RNA	N	N	N	N	N	N	N
ER	Ce	Ce	Ce	Ce	Cy	R Cy	R Cy
AGP	Ce	Ce	Ce	Ce	N Cy	R Cy M	R Cy M
MITO	Ce	Ce	Ce	Ce	N Cy	R Cy	R Cy

(B)

Channel	Colocalization	Granularity	Intensity	Neighbors	Intensity distribution	Size & shape	Texture
DNA	X	X	X	X	X	X	X
RNA	X	X	X	X	X	X	X
ER	X	X	X	X	X	X	X
AGP	X	X	X	X	X	X	X
MITO	X	X	X	X	X	X	X
