# Supplementary material for: Drug-induced cytotoxicity prediction in muscle cells, an application of the Cell Painting assay
Source: PLoS One. 2025 Mar 31;20(3):e0320040. doi: 10.1371/journal.pone.0320040 (PMC11957314; doi:10.1371/journal.pone.0320040)
Supplement: S2 Table — cMAP tau scores of top perturbagen classes (A), top genes (B), and top compound similarities (C). Scores ranging from -100 to + 100, and values over 90 or below -90 are considered of interest for further investigation. Values outside this threshold are outlined in red. Only compound BRD-M64432851 was considered for sunitinib due to the presence of duplicates. Duplicate compound names were truncated for readability. (PDF) [file pone.0320040.s002.pdf]

S2 Table: Connectivity results of sunitinib against the statins ATOR, CERI, SIMV obtained from the cMAP tool ([clue.io]). cMAP *tau* scores of top perturbagen classes (A), top genes (B), and top compound similarities (C). Scores ranging from -100 to +100, and values over 90 or below -90 are considered of interest for further investigation. Values outside this threshold are outlined in red. Only compound BRD-M64432851 was considered for sunitinib due to the presence of duplicates. Duplicate compound names were truncated for readability.

(A)

Perturbagen class	score	ATOR	CERI	SIMV	SUNI
HMGCR inhibitor	99.96	100.00	99.95	99.97	91.51
PI3K inhibitor	99.14	99.11	59.41	99.36	99.16
DNA dependent protein kinase inhibitor	98.92	98.93	62.09	99.35	98.91
SRC inhibitor	98.76	99.53	95.10	99.48	98.04
Serotonin receptor antagonist	98.70	99.14	90.67	99.48	98.27

(B)

Gene symbol	score	ATOR	CERI	SIMV	SUNI
MED4	95.99	95.92	96.05	96.98	81.95
OVOL2	94.71	98.25	93.50	95.91	86.16
C9ORF96	94.39	98.70	94.69	94.09	81.47
MST1R	94.20	98.89	65.27	97.74	90.67
GPR110	93.86	98.66	14.33	95.53	92.20
ATP6V1D	93.15	96.90	86.54	95.07	91.23
PCSK9	92.88	94.71	93.60	92.15	81.86
RXRA	92.79	94.55	8.34	91.03	94.96

(C)

Compound name	score	ATOR	CERI	SIMV	SUNI
RS-17053	99.83	99.86	92.47	99.86	99.79
BIBU-1361	99.77	99.82	99.49	99.79	99.75
wortmannin	99.65	99.68	87.96	99.82	99.61
fluvastatin	99.58	99.89	99.30	99.86	88.73
lovastatin	99.56	99.89	99.33	99.79	85.88
NNC-55-0396	99.56	99.30	71.81	99.82	99.82
CGP-71683	99.53	99.44	61.99	99.79	99.61
