# Supplementary material for: Drug-induced cytotoxicity prediction in muscle cells, an application of the Cell Painting assay
Source: PLoS One. 2025 Mar 31;20(3):e0320040. doi: 10.1371/journal.pone.0320040 (PMC11957314; doi:10.1371/journal.pone.0320040)
Supplement: S3 Table — (PDF) [file pone.0320040.s003.pdf]

S3 Table: List of reagents used for conducting the Cell Painting Assay.

Product	Supplier	Note
DMEM (Dulbecco's modified eagle medium)	ThermoFisher	Supplemented with 20% FBS or 2% HS and 5mL of PenStrep
FBS (Fetal Bovine Serum)	ThermoFisher	
HS (Horse Serum)	ThermoFisher	
HBSS (Hanks Balanced Salt Solution)	ThermoFisher	For cell culture, plate washes
Trypsin-EDTA 1.5x	ThermoFisher	For cell detachment in cell culture
PFA 2%	SigmaAldrich	Cell fixation
HBSS-0.1% Triton X-100	ThermoFisher	For cell permeabilization
