# Supplementary material for: Drug-induced cytotoxicity prediction in muscle cells, an application of the Cell Painting assay
Source: PLoS One. 2025 Mar 31;20(3):e0320040. doi: 10.1371/journal.pone.0320040 (PMC11957314; doi:10.1371/journal.pone.0320040)
Supplement: S4 Table — (PDF) [file pone.0320040.s004.pdf]

**S4 Table: Cell Painting Protocol for C2C12 cells**, adapted from the standard Cell Painting protocol (v3)

<table>
<tr><th>Step</th><th>Action</th></tr>
<tr><td>1.</td><td>Seed 240 wells of a 384-well plate with C2C12 myoblasts. Each occupied well should contain 5,000 myoblasts in 20ul of media.</td></tr>
<tr><td>2.</td><td>Copy the layout of the mother plates (See Mother Plate Formation Protocol) and multiply the number of wells by 4, i.e., each well in the 96-well mother plate is represented by 4 wells in a 2x2 formation on the 384-well plate.
<table>
<tr><th></th><th>1</th><th>2</th><th>3</th><th>4</th><th>5</th><th>6</th><th>7</th><th>8</th><th>9</th><th>10</th><th>11</th><th>12</th></tr>
<tr><td>A</td><td colspan="12">Blank</td></tr>
<tr><td>B</td><td rowspan="6">Blank</td><td rowspan="6">DMSO</td><td colspan="9">Drug 1</td><td rowspan="6">Blank</td></tr>
<tr><td>C</td><td colspan="9">Drug 2</td></tr>
<tr><td>D</td><td colspan="9">Drug 3</td></tr>
<tr><td>E</td><td colspan="9">Drug 4</td></tr>
<tr><td>F</td><td colspan="9">Drug 5</td></tr>
<tr><td>G</td><td colspan="9">Drug 6</td></tr>
<tr><td>H</td><td colspan="12">Blank</td></tr>
</table>
</td></tr>
</table>

| | |
|---|---|
| **3.** | Take 10ul of the lowest concentration of the first toxicant from the 96-well mother plate and add to 90ul of media in a V-bottom 96-well plate (Daughter plate). Take 10ul of this and add to 190ul media in a second 96-well V-bottom plate (Granddaughter plate). Add 5ul of this diluted toxicant to the relevant 4 wells on the 384-well plate, which already contains 20ul. This creates a total drug dilution of 1:1,000 in the well. Repeat for all other wells in the mother plate. No mixing is required. |
| **4.** | Label the plate lid and bottom with tape and incubate at 37°C and 5% $CO_2$ for 72 hours. |
| **5.** | Reconstitute the contents of the PerkinElmer Cell Painting Kit in $ddH_2O$ or DMSO according to the online protocol. If using a 10x384 well kit, make up 50mL of Diluent A. |
| **6.** | Prepare the staining solutions as follows if using a 10x384 well kit:<br>Staining Solution 1 (SS1)<br>● 25mL Diluent A<br>● 16.6ul 641 Mitochondrial stain<br>Staining Solution 2-1 (SS2-1)<br>● 11.75mL Diluent A<br>● 62.5ul Fluor 568 - Phalloidin<br>● 625ul Fluor 488 - Concanavalin A<br>● 62.5ul Hoechst 33342 Nuclear Stain<br>Staining Solution 2-2 (SS2-2)<br>● 11.6875mL Diluent A<br>● 125ul Fluor 555 - WGA<br>● 625ul Fluor 488 - Concanavalin A<br>● 62.5ul Hoechst 33342 Nuclear Stain<br>Store remaining solutions at $-16^{0}$C or lower. |
| **7.** | Aspirate 15ul of media and toxicant from the wells, leaving behind ~10ul of liquid. |
| **8.** | Take 15ml of Staining Solution 1 and dispense into 60 wells of a V-bottom 96-well plate, at 180ul per well. Take 30ul of Staining Solution 1 from each well and add to each well of the 384-well plate. |

| | |
|---|---|
| **9.** | Centrifuge the 384-well plate at 200G for 1 minute. |
| **10.** | Incubate in the dark at 37°C for 30 minutes. |
| **11.** | The following steps must be done with no pauses. Add 10ul PFA to each well. |
| **12.** | Centrifuge plate at 200G for 1 minute. |
| **13.** | Incubate in the dark at room temperature for 20 minutes. |
| **14.** | Aspirate 40ul of liquid, leaving behind ~10ul. |
| **15.** | Add 40ul of HBSS from a trough and aspirate again, leaving behind 10ul. |
| **16.** | Add 30ul of HBSS-0.1% Triton X-100 from a trough. |
| **17.** | Incubate in the dark at room temperature for 10-20 minutes. |
| **18.** | Aspirate 30ul and add 30ul HBSS. Repeat. |
| **19.** | Add Staining Solutions 2-1 and 2-2 into 2 separate V-bottom 96-well plates. There should be 75ul of solution per well. |
| **20.** | Dispense Staining Solutions 2-1 and 2-2 into the 384-well plate so that for every 2x2 quadrant of wells, the top 2 are stained with 30ul of 2-1 and the bottom 2 are stained with 30ul of 2-2. |
| **21.** | Centrifuge the 384-well plate at 200G for 1 minute. |
| **22.** | Incubate the plate at room temperature in the dark for 30 minutes. |
| **23.** | Wash the plate 3 times with HBSS. Do not aspirate the final wash. |
| **24.** | Wrap the plate with foil and store at 4°C until ready for imaging. |
