# Supplementary material for: Drug-induced cytotoxicity prediction in muscle cells, an application of the Cell Painting assay
Source: PLoS One. 2025 Mar 31;20(3):e0320040. doi: 10.1371/journal.pone.0320040 (PMC11957314; doi:10.1371/journal.pone.0320040)
Supplement: S5 Table — (PDF) [file pone.0320040.s005.pdf]

S5 Table: Summary of the best RF feature importances extracted from the global RF model generated on myoblast Harmony dataset, with SMOGN data augmentation.

Feature	RF importance
Nucleus 33342 Profile 5/5	0.06680940014094917
Cytoplasm 488 Profile 3/5 SER-Valley	0.06247169101771283
Cytoplasm 488 Profile 4/5 SER-Edge	0.05841360920583142
Cytoplasm 568 Profile 2/5 SER-Saddle	0.054978450091566045
Cytoplasm Mito Profile 2/5 SER-Saddle	0.05453900507760594
Nucleus 568 Profile 5/5 SER-Saddle	0.05076697479788746
Nucleus Mito Profile 5/5 SER-Bright	0.04512380469990936
Cytoplasm 488 Profile 5/5 SER-Edge	0.04143863106734466
Cytoplasm 488 Profile 3/5 SER-Dark	0.03931834346317344
Nucleus 33342 Radial Mean Ratio SER-Saddle	0.030700559488462783
Cytoplasm 568 Profile 3/5 SER-Saddle	0.028041941658904432
Ring Region Alexa 647 SER Saddle 0 px	0.018162124503203914
Cytoplasm Alexa 568 SER Spot 0 px	0.018075224759560193
Intensity Membrane Resized Alexa 568 Mean	0.016833184721358106
Cell 488 Axial Length Ratio SER-Hole	0.016122908444034495
