# Supplementary material for: Drug-induced cytotoxicity prediction in muscle cells, an application of the Cell Painting assay
Source: PLoS One. 2025 Mar 31;20(3):e0320040. doi: 10.1371/journal.pone.0320040 (PMC11957314; doi:10.1371/journal.pone.0320040)
Supplement: S6 Table — Built on C2C12 Harmony myoblast data, with no data augmentation (PDF) [file pone.0320040.s006.pdf]

S6 Table: Top RF importance of the global RF model. Built on C2C12 Harmony myoblast data, with no data augmentation

Rank	Harmony readout	RF Importance
0	Cytoplasm 488 Profile 4/5 SER-Edge	0,4473647
1	Nucleus 33342 Profile 5/5	0,1604385
2	Cytoplasm Mito Profile 4/5 SER-Bright	0,0828921
3	Cell 488 Radial Mean Ratio SER-Dark	0,0264200
4	Nucleus 33342 Radial Mean SER-Hole	0,0262545
5	Cell Contact Area with Neighbors [%]	0,0086929
6	Nucleus 33342 Axial Small Length SER-Hole	0,0080100
7	Intensity Cytoplasm Alexa 568 Mean	0,0071828
8	Cytoplasm 33342 Profile 3/5 SER-Saddle	0,0063806
9	Cytoplasm 488 Profile 3/5 SER-Saddle	0,0056477
10	Cytoplasm 33342 Profile 3/5 SER-Edge	0,0055301
11	Cell 488 Symmetry 05	0,0045777
12	Cytoplasm 33342 Profile 3/5 SER-Dark	0,0045494
13	Cell 33342 Radial Mean	0,0038113
14	Cytoplasm 488 Profile 3/5 SER-Bright	0,0037020
15	Nucleus 33342 Radial Relative Deviation SER-Hole	0,0034596
16	Nucleus HOECHST 33342 SER Hole 0 px	0,0034007
17	Cell 568 Threshold Compactness 40%	0,0032810
18	Intensity Nucleus HOECHST 33342 StdDev	0,0032727
19	Nucleus 33342 Radial Mean Ratio SER-Bright	0,0030972
20	Cytoplasm 488 Profile 4/5 SER-Hole	0,0029785
21	Cytoplasm Mito Profile 5/5 SER-Edge	0,0027285
22	Cytoplasm 33342 Profile 3/5 SER-Hole	0,0026897
23	Nucleus 33342 Threshold Compactness 30%	0,0026441
24	Cytoplasm 488 Profile 3/5 SER-Dark	0,0024146
25	Nucleus 33342 Radial Relative Deviation SER-Saddle	0,0023839
26	Cytoplasm 568 Profile 4/5	0,0023811
27	Intensity Nucleus HOECHST 33342 Mean	0,0023381
28	Cytoplasm Mito Profile 3/5 SER-Dark	0,0022885
29	Cell Mito Threshold Compactness 60% SER-Edge	0,0022174

30	Cell 568 Threshold Compactness 60%	0,0021979
31	Cytoplasm Alexa 568 SER Bright 0 px	0,0021214
32	Intensity Nucleus Alexa 555 Mean	0,0019119
33	Cytoplasm 568 Profile 5/5 SER-Hole	0,0018901
34	Cell 555 Threshold Compactness 60%	0,0018618
35	Nucleus 33342 Threshold Compactness 30% SER-Bright	0,0018123
36	Cytoplasm 488 Profile 5/5 SER-Bright	0,0018109
37	Cytoplasm 33342 Profile 3/5 SER-Bright	0,0017375
38	Nucleus 33342 Threshold Compactness 30% SER-Saddle	0,0017231
39	Cytoplasm Mito Profile 3/5 SER-Saddle	0,0017180
40	Cytoplasm 33342 Profile 2/5	0,0016423
41	Nucleus Mito Profile 5/5	0,0016375
42	Cell 488 Radial Relative Deviation SER-Dark	0,0015867
43	Nucleus 33342 Threshold Compactness 30% SER-Edge	0,0015068
44	Nucleus 568 Profile 5/5	0,0014436
45	Cell 33342 Radial Mean Ratio SER-Dark	0,0014426
46	Cytoplasm 568 Profile 4/5 SER-Bright	0,0014388
47	Nucleus Mito Profile 5/5 SER-Saddle	0,0014232
48	Cell 555 Threshold Compactness 50%	0,0013928
49	Cell 568 Threshold Compactness 50%	0,0013716
50	Cell Mito Symmetry 03 SER-Spot	0,0013465
51	Nucleus Width [Âµm]	0,0013120
52	Intensity Nucleus HOECHST 33342 CV [%]	0,0012756
53	Cell Roundness	0,0012723
54	Cell 568 Symmetry 05 SER-Dark	0,0012483
55	Cell 488 Threshold Compactness 60%	0,0012320
56	Cell 555 Axial Small Length	0,0012280
57	Cytoplasm 488 Profile 1/5 SER-Dark	0,0012181
58	Cytoplasm 568 Profile 2/5	0,0012156
59	Cytoplasm 33342 Profile 1/5	0,0011890
60	Nucleus Alexa 555 SER Saddle 0 px	0,0011869
61	Cell 488 Symmetry 03	0,0011707
62	Cell 555 Radial Relative Deviation	0,0011512
63	Cytoplasm 488 Profile 2/5	0,0011505
64	Cytoplasm Mito Profile 4/5 SER-Dark	0,0011426

65	Cell 488 Radial Mean Ratio SER-Bright	0,0011308
66	Cell Mito Threshold Compactness 40%	0,0011078
67	Nucleus 33342 Symmetry 05 SER-Saddle	0,0011044
68	Cytoplasm Alexa 488 SER Dark 0 px	0,0010722
69	Ring Region Alexa 647 SER Hole 0 px	0,0010702
70	Cell 568 Symmetry 13 SER-Hole	0,0010050
