# Supplementary material for: Drug-induced cytotoxicity prediction in muscle cells, an application of the Cell Painting assay
Source: PLoS One. 2025 Mar 31;20(3):e0320040. doi: 10.1371/journal.pone.0320040 (PMC11957314; doi:10.1371/journal.pone.0320040)
Supplement: S7 Table — Obtained with the cell count analysis from Harmony data, and from the CellTiter-Glo cell viability assay. (PDF) [file pone.0320040.s007.pdf]

S7 Table: Summary of IC50 and LC50 values. Obtained with the cell count analysis from Harmony data, and from the CellTiter-Glo cell viability assay.

Compound	IC50 viability (nM)	LC50 counts (nM)
DOXO	1150	8
COLC	NA	29
ETOP	31750	120
CERI	760	382
SELU	50860	3905
SUNI	2260	4217
CISP	NA	9106
FIAL	NA	17050
ATOR	32700	18443
SIMV	48270	19064
IMAT	5210	27468
LEFL	NA	28896
MCPP	NA	906000
OLAN	53530	1.00E+06
NELA	NA	6.20E+07
TEBU	32940	NA
CLEV	NA	NA
CLOF	NA	NA
DAPT	NA	NA
DEXA	NA	NA
ETHF	NA	NA
ETRE	NA	NA
EZET	NA	NA
GEMF	NA	NA
HYCQ	NA	NA
IBIP	NA	NA
NCAP	NA	NA
VORI	NA	NA
WURS	NA	NA
ZIDO	NA	NA
