# Supplementary figures and images for: Drug-induced cytotoxicity prediction in muscle cells, an application of the Cell Painting assay
Source: PLoS One. 2025 Mar 31;20(3):e0320040. doi: 10.1371/journal.pone.0320040 (PMC11957314; doi:10.1371/journal.pone.0320040)

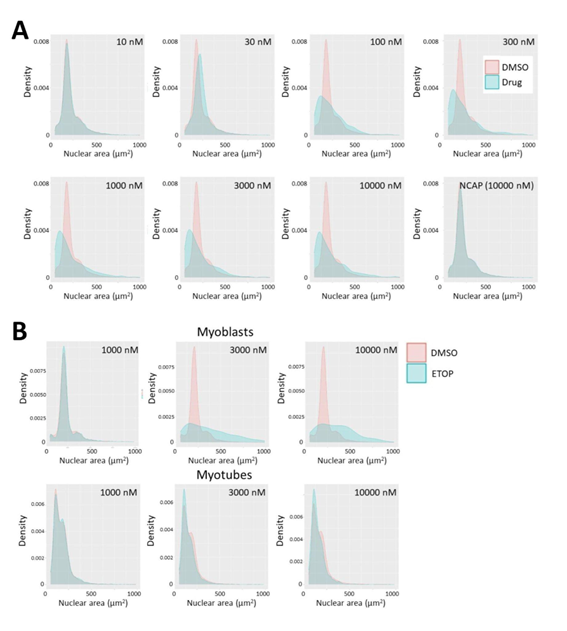

Supplement: S1 Fig — Aminocaproic acid (NCAP) is a control drug. (B) Concentration dependent changes in myoblast and myotube nuclear area induced by Etoposide (ETOP). The figure can be reproduced from 220412 and 220425 data sets using the accompanying code in S1 Appendix. (PNG) [file pone.0320040.s008.png]

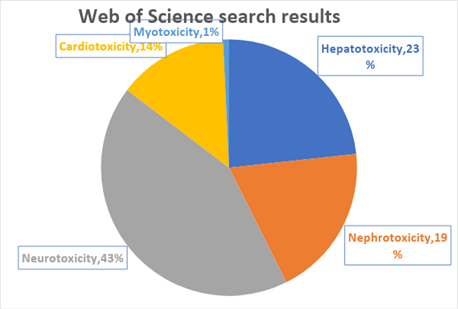

Supplement: S2 Fig — Analysis performed on August 1st 2023. Results given in percentage of total documents yielded with all queries. Keywords were entered as displayed in lowercase, and no synonyms were considered. Search conducted in the WoS Core Collection only, including all document types. (PNG) [file pone.0320040.s009.png]

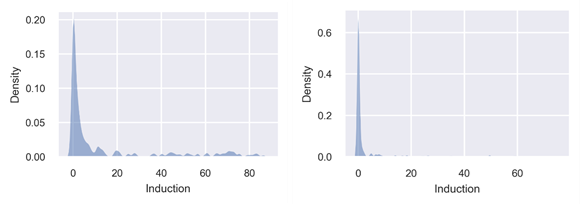

Supplement: S3 Fig — (PNG) [file pone.0320040.s010.png]

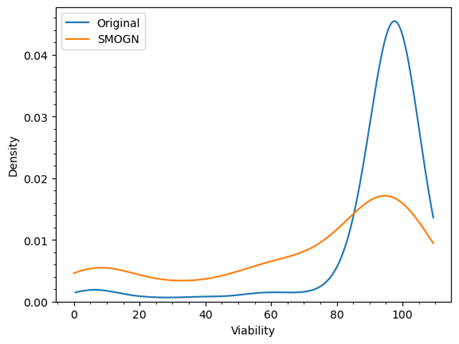

Supplement: S4 Fig — Treatment-level data, before data augmentation (blue, Original) and after data augmentation using the SMOGN algorithm (orange, SMOGN). (PNG) [file pone.0320040.s011.png]

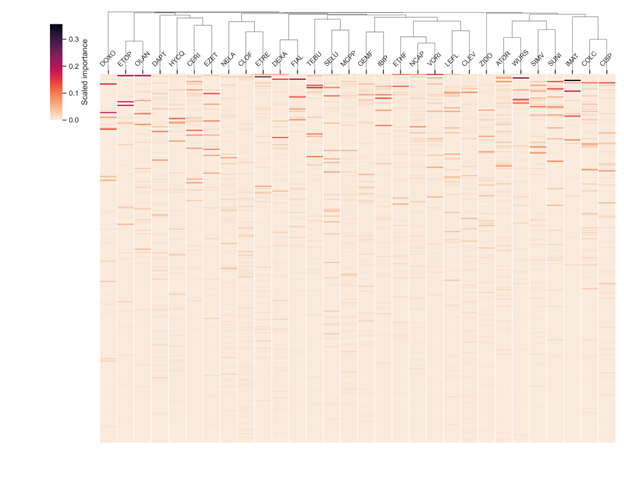

Supplement: S5 Fig — Features are ranked in descending order by their median rank of importance across all models (most important features are at the top of the heatmap). Clustering is performed using single-linkage and correlation metric. The lower half of the heatmap containing less prominent features was cropped for readability. (PNG) [file pone.0320040.s012.png]

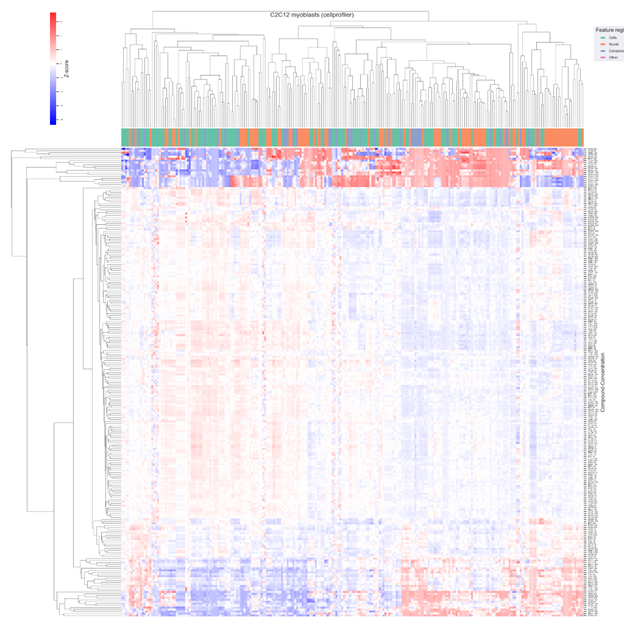

Supplement: S6 Fig — All profiles are represented, no induction filter. Hierarchical clustering of treatments and features computed with average-linkage on Pearson correlations. Z-scores clipped to ± 8 MAD for increased readability and color scaling. (PNG) [file pone.0320040.s013.png]

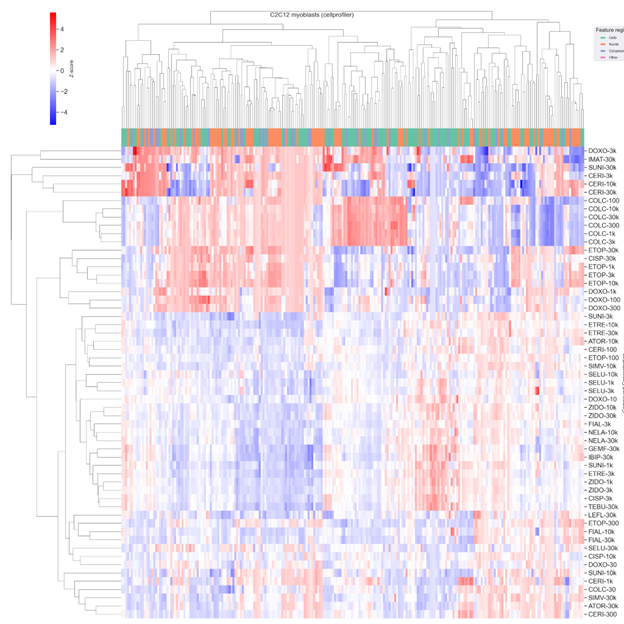

Supplement: S7 Fig — Profiles with induction > 0.2 are represented. Hierarchical clustering of treatments and features computed with average-linkage on Euclidean distances. Z-scores clipped to ± 8 MAD for increased readability and color scaling. (PNG) [file pone.0320040.s014.png]
