# Supplementary material for: Drug-induced cytotoxicity prediction in muscle cells, an application of the Cell Painting assay
Source: PLoS One. 2025 Mar 31;20(3):e0320040. doi: 10.1371/journal.pone.0320040 (PMC11957314; doi:10.1371/journal.pone.0320040)
Supplement: S1 Appendix — (PDF) [file pone.0320040.s015.pdf]

S1 Appendix: Analysis of Colchicine data

Plate layout

Plate number:[DRUG1...DRUG6]

1:['NCAP','ATOR','CERI','CLOF','COLC','DAPT'],

2:['DEXA','DOXO','EZET','HYCQ','LEFL','SELU'],

3:['CLEV','ETOP','FIAL','GEMF','IBIP','MCPP'],

4:['ETHF','ETRE','NELA','SIMV','VORI','WURS'],

5:['CISP','IMAT','OLAN','SUNI','TEBU','ZIDO']}

Uploading raw data files

1. Download folder contain experiment.
2. Unzip folder.
3. Rename file "Objects_Population – valid objects" appropriately (e.g. Plate1_blasts_[date].txt)
4. Move file to appropriate directory.

Myoblasts

```
require(tidyverse)
require(ggplot2)
data<- read.delim("Plate1_220425.txt",
         header = TRUE,
         sep = "\t",
         dec = ".",
         skip = 9)

#Select data on nuclear area.
```

```
data1<-select(data, Row, Column, Compound, Concentration,valid.objects...Nucleus.Area..µm..)
>head(data1)

#rename DMSO label so "0" conc can be used.
recoded<-data1 %>%
mutate(Compound = recode(Compound, DMSO = "Colc",DRUG5 = "Colc"))

# Change column heading of "valid.objects...Nucleus.Area..µm.." to "Area"

renamed<-recoded %>%
  rename(Area = valid.objects...Nucleus.Area..µm..)
head(renamed)

#remove outliers

renamed<-subset(renamed, Area >50)

renamed<-subset(renamed, Area <1000)

a<-subset(renamed, Compound == "Colc")

a %>%

  #na.omit(Concentration). Unexplained problem with plotting NA values...

    mutate(Concentration = factor(Concentration,levels=c("0","100"))) %>%

  na.omit(Concentration) %>%

    ggplot(aes(x = Area, fill = Concentration, colour = Concentration))+

    geom_density(alpha = 0.2)

theme_bw()

write.csv(a, "Colc_only.csv")
```

Myotubes

```
require(tidyverse)

require(ggplot2)
```

```
data<- read.delim("Plate1_tubes_220425.txt",
                  header = TRUE,
                  sep = "\t",
                  dec = ".",
                  skip = 9)
head(data1)
tail(data1)

#rename DMSO label so "0" conc can be used.
recoded<-data1 %>%
 mutate(Compound = recode(Compound, DMSO = "Colc",DRUG5 = "Colc"))
# Change column heading of "valid.objects...Nucleus.Area..µm.." to "Area"
renamed<-recoded %>%
  rename(Area = valid.objects...Nucleus.Area..µm..)
#remove outliers
renamed<-subset(renamed, Area >50)
renamed<-subset(renamed, Area <1000)
head(renamed)
a<-subset(renamed, Compound == "Colc")
a %>%
  #na.omit(Concentration). Unexplained problem with plotting NA values...
  mutate(Concentration = factor(Concentration,levels=c("0","100"))) %>%
  na.omit(Concentration) %>%
  ggplot(aes(x = Area, fill = Concentration, colour = Concentration))+
  geom_density(alpha = 0.2)
theme_bw()
```
