# Supplementary material for: Drug-induced cytotoxicity prediction in muscle cells, an application of the Cell Painting assay
Source: PLoS One. 2025 Mar 31;20(3):e0320040. doi: 10.1371/journal.pone.0320040 (PMC11957314; doi:10.1371/journal.pone.0320040)
Supplement: Attachment — Submitted filename: Reply to individual comments.pdf [file pone.0320040.s016.pdf]

Reviewer #1:

Unfortunately, most of the comments and suggestions from Reviewer #1 were of no help to further improving our manuscript, notably because the discussed subject is not post-traumatic osteoarthritis (PTOA), as mentioned in the reviewer's comments. This suggests a potential misunderstanding, miscommunication regarding the scope of our work, or a mixup with another manuscript may have occurred.

Clearly state the research objective or hypothesis.

The research objective and hypothesis are thoroughly stated.

1. ***Provide a brief background on post-traumatic osteoarthritis (PTOA), its prevalence, and the need for understanding its molecular mechanisms.***
2. **Describe the methods used for inducing PTOA in the mouse models.**
3. **Discuss the significance of using PTOA-susceptible and PTOA-resistant mouse strains in the study.**

Provide a comprehensive review of the relevant literature on PTOA and immune cell involvement.

This comment is not relevant in the context of our study.

Post-traumatic osteoarthritis (PTOA) is not the subject of this research.

Discuss the potential implications of the observed differences in monocyte and macrophage subpopulations between MRL and B6 joints.

No mention of monocytes, macrophage populations, MRL or B6 joints were made in the manuscript.

Discuss the role of CD206+ macrophages and Trem2 receptor in PTOA development or resolution.

Post-traumatic osteoarthritis (PTOA) is not the subject of this research.

Consider comparing the findings with previous studies in the field.

Discuss the potential mechanisms underlying the enhanced clearance of debris and apoptotic cells in MRL injured joints.

Post-traumatic osteoarthritis (PTOA) is not the subject of this research.

Discuss the limitations of the study, such as the use of animal models and the extrapolation to human PTOA.

Post-traumatic osteoarthritis (PTOA) is not the subject of this research.

Provide a clear conclusion summarizing the key findings and their implications.

A conclusion was already presented in the manuscript, but was rewritten for clarity.

Ensure that the manuscript adheres to the journal's formatting and citation guidelines.

We made sure that the manuscript strictly followed the PLoS: One formatting and citation policies.

Include a discussion on the potential clinical relevance of the findings and their impact on PTOA treatment strategies.

Post-traumatic osteoarthritis (PTOA) is not the subject of this research.

Consider including visualizations or figures to illustrate the single-cell RNA sequencing results.

No single-cell RNA sequencing was performed in this research study.

Discuss the potential future research directions based on the study findings.
Consider discussing the limitations and challenges of translating the findings to human PTOA.

Post-traumatic osteoarthritis (PTOA) is not the subject of this research.

Provide a comprehensive list of references to support the research background and contextualize the findings.

Multiple references supporting the research background and our findings are provided.

Consider the potential implications of the study for personalized medicine approaches in PTOA management

Post-traumatic osteoarthritis (PTOA) is not the subject of this research.

Reviewer #2

Limited Novelty: While the use of in silico toxicity prediction and Cell Painting techniques is of interest, the presented findings do not provide significant new insights beyond what has already been reported in the literature.

There is no data on muscle toxicity. Cell context/identity is often central to determine responses to toxicants, affected by the cell-specific expression of targets, and toxifying/de-toxifying metabolic pathways and variable levels of cellular defenses.

Moreover, in this manuscript we report the first instance of phenotypic cell profiling of C2C12 cells in the litterature. To our knowledge, Cell Painting or other kind of profiling assay has never been conducted before this study on this cell line.

Insufficient Contextualization: The introduction does not adequately establish the importance and relevance of in silico toxicity prediction and the limitations of current approaches. It is crucial to provide a comprehensive overview of the challenges in toxicology testing and the potential benefits of integrating computational methods.

The introduction has been revised to highlight the relevance and the importance of in silico prediction of toxicity in skeletal muscle cells.

Lack of Comparative Analysis: The article does not compare the findings of the study with previous studies or existing knowledge in the field. A more thorough comparison would help to assess the significance and contribution of the current study.

Due to the lack of other phenotypic profiling studies of C2C12 cells, comparative analysis could not be performed in the current state of research, and comparison with data from other cell types or cell lines is out of the scope of this study.

Incomplete Methodological Description: The article lacks a detailed description of the computational approaches used for in silico toxicity prediction and the specific parameters employed. It is important to provide sufficient information on the data integration, feature extraction, and model training processes to ensure the validity and reproducibility of the results.

All the computational approaches used in this study have all been thoroughly documented and described in the Methods section of our manuscript. If any specific methodology is unclear, we are open to provide any additional detail that is not currently present in the manuscript. However, due to the lack of specific mention in the reviewer's comment, it is unclear for us to know which methodological approach is lacking detail.

Inadequate Discussion of Functional Implications: The article does not sufficiently discuss the functional implications of using a myoblast cell line for toxicity assessment and the relevance of these findings to broader toxicological endpoints. A more comprehensive discussion of the functional aspects would enhance the understanding of the findings.

The C2C12 cell line provides a robust, reproducible model of both myoblasts (a stem-cell like cell) involved in repair and regeneration and myotubes, a differentiated muscle cell type that expresses muscle specific genes and executes muscle specific processes not modeled in other in vitro cell painting studies. A detailed discussion on the relevance of the findings is now provided in the manuscript as suggested.

Insufficient Literature Review: The article lacks a thorough review of the existing literature on in silico toxicity prediction, Cell Painting techniques, and their applications in toxicology. A comprehensive review would provide a broader context for the presented findings.

The literature review in the introduction was greatly revised to better represent the state of research in the field.

Lack of Clarity in Results Presentation: The presentation of results and findings in the article is unclear and lacks proper organization. The information provided should be presented in a logical and concise manner to facilitate understanding.

The description of results was clarified, and additional analysis was conducted to support our claims, with for instance a study underlying the changes in myoblast nuclei size when treated with colchicine.

Incomplete Discussion of Limitations: The article does not adequately address the limitations of the study, such as the choice of the myoblast cell line, the representativeness of the panel of myotoxicants, or the generalizability of the findings. It is important to acknowledge and discuss these limitations to provide a balanced interpretation of the results.

A section dedicated to the limitations of our approach and techniques has been added. The discussion section of the manuscript was also completely reworked for more accurate language and clarity.
