# Supplementary material for: Drug-induced cytotoxicity prediction in muscle cells, an application of the Cell Painting assay
Source: PLoS One. 2025 Mar 31;20(3):e0320040. doi: 10.1371/journal.pone.0320040 (PMC11957314; doi:10.1371/journal.pone.0320040)
Supplement: Attachment — Submitted filename: ROUND2 - ReplyToComments.pdf [file pone.0320040.s017.pdf]

Review Comments to the Author

Reviewer #3: The revised manuscript is significantly improved but there are some issues with the formatting and content.

Lines 130-134 and 230-234 seem to be the same but the references are different.
C2C12 cell line introduction section should be placed in the in Introduction chapter.

We thank the reviewer for this useful comment. The introduction of the C2C12 cell line has been moved to the Introduction section as suggested. The mismatch of references was due to a mixup between the tracked changes version and the final manuscript version, which has been corrected.

References are missing in line 83, 84 and 89.

We thank the reviewer for catching this reference issue. A mixup between the tracked changes version and the final manuscript version was identified, and this mistake has been corrected in this new resubmission.

Page 10 of the revised manuscript, lines 237 and 239: there are no Figure 2A, 2C, and 2D. I guess the corresponding sentences refer to Figure 1. Figure 1B is not cited anywhere in the manuscript.

Figure 2 has been renamed in the final manuscript, and the identified issue was due to the same mixup of the tracked changes version, which has been corrected.

Lines 463 and 468 in the discussion, what number does the "xxx" correspond to?

We want to thank the reviewer for pointing out that mistake. This figure reference placeholder was still present in the tracked changes version of the previous submission due to a mixup in versioning. This has not been corrected. We apologize for the inconvenience.

Reviewer #5: Nothing much. The revised paper and images (may be, change the graph colors and designs - the excel designs are too obvious - just a personal recommendation) have enough clarity.
All the sections have been revised thoroughly.

We thank the reviewer for their positive feedback and helpful suggestion regarding the graph design. We have revised our figures using a more professional visualization approach, moving away from Excel styling to enhance the clarity and professional appearance of our results, now using Python.

Reviewer #6: The authors have presented an application of the cell painting assay to evaluate drug-induced mytotoxicity in C2C12 cells, both as myoblasts and having

undergone differentiation to . While the application of the technique is well intended in the field of toxicology, there are several concerns regarding the methodology, particularly the machine learning and statistical analysis.

In a nutshell, the sources stated in the data availability statement (Zenodo and GitHub) are not accessible and therefore cannot be evaluated during peer-review. This is a very strong ground for rejection, but as a courtesy I am still willing to review the shared material and give the authors a chance to share the remaining data at the time of revision. Please find below the concerns that need to be addressed in order to prepare the submission for publication:

1. In Figure 1, please include the colour mapping in the figure itself. The images in the figure also lack a scale. Please justify your reason to highlight the 100 mM exposure of colchicine in the manuscript text associated with the figure.

We thank the reviewer for this comment. Color coding of the different image channels has been added to Figure 1, as well as a scale to help the representation of object size. COLC 100nM was selected as it is the lowest concentration where a significant decrease in cell count was observed. An explanation has been added to the manuscript text.

2. Please prepare a supplementary table/spreadsheet with all the identified features following analysis in Harmony and CellProfiler (lines 114-115).

The supplementary table (Table S1) was included to summarize the different types of features extracted with the two feature extraction pipelines, obtained with Harmony and Cell Profiler. Unfortunately, due to the extensive number of features calculated with each feature extraction pipeline, we are unable to include an exhaustive list of features. With this solution, we hope the table summary will help the understanding of the different types of readouts studied in this manuscript.

3. Please make available the image analysis pipeline for Harmony and CellProfiler as supplementary data (say on GitHub).

We thank the reviewer for this important suggestion. The Cell Profiler pipelines, the Jupyter notebooks as well as the Python scripts used for the analysis have now been made available on the GitHub repository and are freely accessible. Due to the close source nature of the Harmony building blocks pipeline, we are not able to share it as is and would require more expertise to extract it from Harmony. We will however make it available on GitHub as soon as we are able to. We are sorry for this inconvenience.

4. In line 121, how were the optimal phenotypes selected (what criteria did you use for this)? This requires further elaboration. Where can I find the output for the analysis that was used to make this selection?

Unfortunately no in-depth study was conducted for selecting the Robust MAD profiles, but a qualitative analysis on the heatmaps of the different profiles, as well as compound separation on several PCAs showed that profiles obtained with Zscore or MinMax normalization were more subject to inter-plate variability compared to the ones obtained with the RobustMAD method.

5. Please include significance values (adjusted p values, R2 values etc.) wherever you have claimed significance in the results section text of the manuscript. The reader must not always have to look at the linked figure/supplementary data to find this.

The word "significant" was sometimes wrongly used in the context of the manuscript, where no significance tests were actually conducted. We have rephrased our claims to reflect this information, and we have conducted statistical tests when possible. Please accept our apologies, as English is not the mother tongue of all the authors. Additionally, cell count data has now been normalized to the vehicle controls to allow easier comparison to cell viability data. Statistical tests have been conducted for cell viability and cell count data. For each concentration, significance has been tested against the vehicle controls and symbols were added on top of each bar to indicate the significance level.

6. In Figure 2, can the cell count values also be plotted as percentage of control like the viability dataset? This will make it easier than comparing absolute to normalised median values in the same plot. Please also add significance symbols for at least the comparison with the control for each readout.

We thank the reviewer for this important comment. Consequently, cell count data has been normalized to the vehicle controls to allow easier comparison to cell viability data, and better readability of the figure. Statistical tests have also been conducted for each concentration against the vehicle controls to assess statistical significance, and symbols were added on top of each bar. We also reverted our figure to a barplot format to make the quantity of information easier to read.

7. In line 138, you mention the ATP based assay for cell cytotoxicity. It would be nice to discuss why you didn't go for other more well-established assays in toxicology, such as the Alamar blue or MTS assays. Do any of the included drugs in the panel have an impact on energy/ATP metabolism, especially glycolysis which is more-prominent in-vitro?

Thank you for this insightful question about our choice of cell viability assay. Our choice quickly ported to CellTiter-Glo, as it is known that ATP measurement provides a rapid and sensitive indicator of metabolically active cells. Existing studies are showing its ability to detect subtle changes in cell health before morphological changes become apparent (Crouch et al., 1993, DOI 10.1016/0022-1759(93)90011-u). While Alamar Blue and MTS assays are indeed well-established, ATP assays often demonstrate higher sensitivity, particularly when assessing early cytotoxic effects. We indeed suspect that some of our drugs may impact ATP

metabolism, especially in the mitochondria, which is another motivation to use an ATP-based cell viability assay in this study to capture these effects.

8. In line 152 – 154, I cannot locate the dataset for the results that have been described. You highlight certain phenotypic changes here, but where are the supporting images?

A reference to Figure 1 was missing from the manuscript text, and was hereby added thanks to this suggestion.

9. In Figures 3 and 4, please include the actual R2 value with each plotted bar. Also improve the readability of the y axis labels with the plotted bars (you can add axis delimiters).

We thank the reviewer for this suggestion which will allow a better readability of our figure. All R2 values have been added to each bar plotted in this figure.

10. For the random forest machine learning, have you calculated the AUC of ROC, sensitivity, specificity and accuracy for each model? I cannot find the code used to generate the models as well.

While ROC AUC, sensitivity and specificity are indeed crucial metrics that must be monitored when building classification models, they cannot be computed when building regression models, such as in this study. That is why we chose to assess the performance of the regression models by means of the R-squared, and the MSE, not shown in the results as they resulted in equal model rankings. The code used to generate the global and individual models has been submitted to the GitHub repository.

11. Please identify the top features of the dataset that contribute to the prediction models, particularly to the combined model. For example, this can be done by calculating SHAP scores (https://doi.org/10.1038/s42256-019-0138-9) that can identify important features from the random forest models that were generated in the study.

We want to thank the reviewer for this suggestion regarding SHAP analysis. SHAP values are robust and well-known indicators for the contribution of each feature to ML models. However in the present study, feature contribution for each model has already been assessed via computation of the feature importance extracted during model building. Our primary goal with this approach was to assess the global importance of features across the entire dataset, which is what RF feature importance already provides. While SHAP values can provide an additional perspective through local interpretability of the features, we consider that our current analysis already satisfies the research questions and identifies the key phenotypic properties associated with cell viability.

12. I would recommend to move Figure 5 to the supplementary and instead generate a new figure from the same dataset that clearly visualises the top 30 – 50 features. The current

figure is good at demonstrating the clustering of the drug responses, but not much more beyond that. The top features should be then discussed as well.

We want to thank the reviewer for this comment. Accordingly, the original figure has been moved to supplementary data, and a new Figure using the same dataset has been replaced as Figure 5, where the top 30 features according to their importance in individual models are analyzed and clustered.

13. In lines 190 – 191, you state that there is no correlation between cell count, viability and the resulting clusters. Please mention which results you are referring to when making this statement as it is unclear.

This observation taken from the original Figure 5 were an attempt at proving that important features in individual compound models and the significant decrease in count or viability were not correlated. This part of the manuscript is not relevant anymore with the change in Figure 5, and has been discarded.

14. Please prepare a supplementary table/spreadsheet with all features that were identified in the random forest machine learning analysis, for individual as well as combined models.

Due to the important size of the tables containing the feature importance of each model, we have added the table of Random Forest feature importances computed for the global model built with Harmony features in supplementary materials. All features with an importance above 0.001 are displayed in this table, which make up 71 out of 310 total Harmony features.

15. Have you performed any exploratory analysis with PCA/OPLS-DA to identify important features that have an impact on the dataset? Have you compared this and other regression models with the random forest machine learning?

Multiple linear regression, polynomial regression and simpler decision trees were also tested in preliminary studies, but Random Forests yielded the best results in this use case. Several PCAs have been conducted for visualization purposes, but the contribution of each phenotypic property to the principal components have not been studied here. This idea could be of interest for such models in future experiments, but we are confident that the RF feature importance used in the present study is a reliable metric to assess the impact of features on the predictive models.

16. Please justify your choice of random forest machine learning in the discussion. Have other models been previously applied to cell painting assays? How does your model perform compared to other studies (independent of cell type, only concerning cell painting to predict features).

Random Forests was chosen after a comparison to linear and polynomial regression models, which didn't give any satisfactory results. To our knowledge, this study is the first occurrence of a Machine Learning model leveraging Cell Painting features to predict cell viability.

17. I cannot locate the data on the basis of which the results in lines 214 – 218 have been described. This needs to be properly tagged to the corresponding dataset and further elaborated upon.

The model performance for C2C12 myotubes results are given in Figures 3 and 4. A reference to these figures has been added to the manuscript to improve the clarity of this paragraph.

18. I am uncertain with the statement in line 228. Please include the analysis in the supplementary data without the selected 20% induction threshold (please clarify in lines 224 – 225 how you arrived at this) to support this statement and/or rewrite in a more factual manner.

The corresponding text was rephrased for clarity, and a reference to previous works was added. The 20% induction threshold was chosen empirically and also motivated by previous works, such as in Schneidewind et al 2020 (DOI 10.1002/cbic.202000381), in which phenotypes are filtered for induction values between 17% and 37%. We hence believe that our 20% lower threshold is a good compromise between filtering out noisy phenotypes, and maintaining a good signal from the meaningful ones.

19. You have mentioned Figure 6 in the results (line 223). Please describe in the text what you interpret from the included figure.

The interpretation of the results displayed in Figure 6 has been consolidated, and were indeed lacking depth in the previous version of the manuscript. We want to thank the reviewer for pointing out this omission.

20. It is unclear where the features highlighted in Table 2 come from in terms of the applied machine learning (from only Harmony, only CellProfiler or both). Please clarify in the text.

Clarification on the origin of the dataset has been added to the text and to the table legend. Harmony data was used to produce these results.

21. In line 233, are you referring to Figure S3 instead of S4? Please check.

We are grateful to the reviewer for catching this typo. The text was indeed referring to Figure S3, and not Figure S4. This reference has been corrected in the manuscript.

22. In line 237, you mention that you calculated Euclidean distances as well, but with "poor

results”. Please provide these results in the supplementary and indicate the nature of the results in more detail.

The section dedicated to the Euclidean distance-based hierarchical clustering has been rephrased to reflect the exclusion criteria, and the Euclidean based figure was added as a supplementary Figure (S6).

23. Supplementary Table 2 has not been attributed in the text of the results section (paragraph from line 247).

A reference to Table S2, which contains the results of the connectivity analysis using the cMAP platform has been added to the manuscript paragraph. We thank the reviewer for pointing out this oversight.

24. Please indicate the results from which the statement in line 260 has been mentioned.

We thank the reviewer for this comment. The statement from line 260 regarding profile similarities between NELA and ZIDO has hence been removed, which we deemed irrelevant after consideration.

25. In line 325, do you mean to state Figure S4 instead of S3? Please check.

We are grateful to the reviewer for catching this typo. The text was indeed referring to Figure S4, and not Figure S3. This reference has been corrected.

26. The limitations section in the discussion is introducing additional results that are not in the results section of the manuscript (lines 320 – 325). This should be moved to the results section and restructured appropriately. Additionally, provide information about this analysis in the methodology as well.

We want to thank the reviewer for this insightful comment. The SMOGN data generation section has been moved from the discussion to the results section accordingly with modifications to the presented results and the methodology section.

27. In the methodology section, please provide more information of the drug library. What was the inclusion/exclusion criteria? State the reasoning behind the choice of the drug library.

Information on the methodology and reasoning used to select the compounds of the Toxifate library has been added to the manuscript text

28. The cell painting protocol needs to be further elaborated in the methodology section as it is a key method of the paper. Simply citing previous work with the application is not sufficient. Also, include a detailed list of reagents used for the staining as a table in the supplementary data. In general, it is recommended to provide catalogue numbers along

with the supplier details for each reagent and version number for each tool in the methodology section.

We want to thank the reviewer for this insightful comment that will allow a better repeatability of this experiment. Consequently, additional information regarding the reagents used for the Cell Painting assay has been added to the methodology section. Additionally, the full protocol of the CPA has been added to supplementary data, as well as the list of reagents used.

29. In the methodology section, the data analysis part requires further elaboration. State the different statistical tests used, post-hoc analysis conducted, significance thresholds and other relevant information. Do this for each analysis that has been included for the manuscript.

The data analysis paragraph in the methodology section of the manuscript has been consolidated with the reviewer's suggestions. Information about the statistical tests has been added to the methodology section.

30. There is also a need to further elaborate on the model building in the methodology section. How many trees were generated, how many iterations were made, what were the significance thresholds, and were there any corrections for multiple testing made?

Additional information on RF model building was added to the Materials and Methods section, highlighting the parameters of the algorithm. No significance thresholds were used for this analysis, and multiple tests were not conducted. Details on the parameters of the Random Forests algorithm such as number of trees, static seed, splitting criteria, were added.
