# Supplementary material for: Drug-induced cytotoxicity prediction in muscle cells, an application of the Cell Painting assay
Source: PLoS One. 2025 Mar 31;20(3):e0320040. doi: 10.1371/journal.pone.0320040 (PMC11957314; doi:10.1371/journal.pone.0320040)
Supplement: Attachment — Submitted filename: ROUND3 - ReplyToComments.pdf [file pone.0320040.s018.pdf]

Reply to the reviewers comments

Reviewer #3: My comments and questions were addressed in a satisfactory way. I have no further questions to the authors and recommend the acceptance of the manuscript.

Reviewer #6: I would like to thank the authors for addressing the raised concerns in the previous round of review. I am satisfied with the responses provided by the authors and appreciate the improvements in the manuscript. I now only have some minor recommendations, after which I will deem the manuscript acceptable for publication:

1. In Figure 1, please increase the font size of the scale bar label and mention this in the figure legend as well.
We want to thank the reviewer for this feedback. The font size of the scale label has been significantly increased, which improves figure readability, and a mention to the caption has been added.

2. In Figure 2, the significance symbols are overlapping with the error bars in some of the plots, making it difficult to interpret. Please correct the placement of these and include what they indicate in the figure legend.
We thank the reviewer for catching this inconsistency in the symbol placement, which hindered the legibility of the figure. This overlap has been now corrected, and the meaning of the symbols has been explained in the caption.

3. Please use a uniform style to refer to the figures in the manuscript text (such as Fig 1 or Figure 1). Please also name the supplementary figures/tables consistently in the text and supporting information document (such as Figure S1 or Supplementary Figure 1).
The figure and table naming conventions have now been harmonized in the manuscript text and in the supplementary information document.

4. In the supporting information document, I recommend placing each supplementary table/figure on a separate page, so that there are no cropping issues (check Supplementary Figure 3) or issues with following the table/figure legends.
We want to thank the reviewer for this comment improving the legibility of the supplementary data. This improvement has been implemented and page breaks have been added for improved readability.

5. I cannot locate the LC50 information in the supporting data as stated in line 132. Please look into this and update as required.
We want to thank the reviewer for catching this oversight in the supplementary data. This has now been corrected, and the LC50 information is now available in S8 Table.
