# Supplementary material for: Drug-induced cytotoxicity prediction in muscle cells, an application of the Cell Painting assay
Source: PLoS One. 2025 Mar 31;20(3):e0320040. doi: 10.1371/journal.pone.0320040 (PMC11957314; doi:10.1371/journal.pone.0320040)
Supplement: Peer review history

# Author comment: Author response to Decision Letter 0

**DOI:** 10.1371/journal.pone.0320040.r001

**Transfer Alert**

This paper was transferred from another journal. As a result, its full editorial history (including decision letters, peer reviews and author responses) may not be present.

6 Mar 2024

# Peer review: Decision Letter 0

**DOI:** 10.1371/journal.pone.0320040.r002

**Authors:** Zhiwen Luo

29 Apr 2024

PONE-D-24-09028Drug-induced cytotoxicity prediction in muscle cells, an application of the Cell Painting assayPLOS ONE

Dear Dr. Lambert,

Thank you for submitting your manuscript to PLOS ONE. After careful consideration, we feel that it has merit but does not fully meet PLOS ONE’s publication criteria as it currently stands. Therefore, we invite you to submit a revised version of the manuscript that addresses the points raised during the review process.

Please submit your revised manuscript by Jun 13 2024 11:59PM. If you will need more time than this to complete your revisions, please reply to this message or contact the journal office at plosone@plos.org . When you're ready to submit your revision, log on to https://www.editorialmanager.com/pone/ and select the 'Submissions Needing Revision' folder to locate your manuscript file.

Please include the following items when submitting your revised manuscript:

- A rebuttal letter that responds to each point raised by the academic editor and reviewer(s). You should upload this letter as a separate file labeled 'Response to Reviewers'.
- A marked-up copy of your manuscript that highlights changes made to the original version. You should upload this as a separate file labeled 'Revised Manuscript with Track Changes'.
- An unmarked version of your revised paper without tracked changes. You should upload this as a separate file labeled 'Manuscript'.

If you would like to make changes to your financial disclosure, please include your updated statement in your cover letter. Guidelines for resubmitting your figure files are available below the reviewer comments at the end of this letter.

If applicable, we recommend that you deposit your laboratory protocols in protocols.io to enhance the reproducibility of your results. Protocols.io assigns your protocol its own identifier (DOI) so that it can be cited independently in the future. For instructions see: https://journals.plos.org/plosone/s/submission-guidelines#loc-laboratory-protocols . Additionally, PLOS ONE offers an option for publishing peer-reviewed Lab Protocol articles, which describe protocols hosted on protocols.io. Read more information on sharing protocols at https://plos.org/protocols?utm_medium=editorial-email&utm_source=authorletters&utm_campaign=protocols .

We look forward to receiving your revised manuscript.

Kind regards,

Zhiwen Luo

Academic Editor

PLOS ONE

Journal Requirements:

When submitting your revision, we need you to address these additional requirements.

1. Please ensure that your manuscript meets PLOS ONE's style requirements, including those for file naming. The PLOS ONE style templates can be found at 

https://journals.plos.org/plosone/s/file?id=wjVg/PLOSOne_formatting_sample_main_body.pdf and 

https://journals.plos.org/plosone/s/file?id=ba62/PLOSOne_formatting_sample_title_authors_affiliations.pdf

2. Please note that funding information should not appear in any section or other areas of your manuscript. We will only publish funding information present in the Funding Statement section of the online submission form. Please remove any funding-related text from the manuscript.

3. Thank you for stating the following financial disclosure: 

   "This study is part of a project "Toxifate" funded by the European Union’s Horizon 2020 research and innovation program under grant agreement No 955830 "

Please state what role the funders took in the study.  If the funders had no role, please state: "The funders had no role in study design, data collection and analysis, decision to publish, or preparation of the manuscript." 

If this statement is not correct you must amend it as needed. 

Please include this amended Role of Funder statement in your cover letter; we will change the online submission form on your behalf.

4. When completing the data availability statement of the submission form, you indicated that you will make your data available on acceptance. We strongly recommend all authors decide on a data sharing plan before acceptance, as the process can be lengthy and hold up publication timelines. Please note that, though access restrictions are acceptable now, your entire data will need to be made freely accessible if your manuscript is accepted for publication. This policy applies to all data except where public deposition would breach compliance with the protocol approved by your research ethics board. If you are unable to adhere to our open data policy, please kindly revise your statement to explain your reasoning and we will seek the editor's input on an exemption. Please be assured that, once you have provided your new statement, the assessment of your exemption will not hold up the peer review process.

Additional Editor Comments:

Authors should respond to reviewer comments point by point

[Note: HTML markup is below. Please do not edit.]

Reviewers' comments:

Reviewer's Responses to Questions

**Comments to the Author**

1. Is the manuscript technically sound, and do the data support the conclusions?

The manuscript must describe a technically sound piece of scientific research with data that supports the conclusions. Experiments must have been conducted rigorously, with appropriate controls, replication, and sample sizes. The conclusions must be drawn appropriately based on the data presented. 

Reviewer #1: Yes

Reviewer #2: Partly

**********

2. Has the statistical analysis been performed appropriately and rigorously? 

Reviewer #1: Yes

Reviewer #2: Yes

**********

3. Have the authors made all data underlying the findings in their manuscript fully available?

The PLOS Data policy requires authors to make all data underlying the findings described in their manuscript fully available without restriction, with rare exception (please refer to the Data Availability Statement in the manuscript PDF file). The data should be provided as part of the manuscript or its supporting information, or deposited to a public repository. For example, in addition to summary statistics, the data points behind means, medians and variance measures should be available. If there are restrictions on publicly sharing data—e.g. participant privacy or use of data from a third party—those must be specified.

Reviewer #1: Yes

Reviewer #2: No

**********

4. Is the manuscript presented in an intelligible fashion and written in standard English?

PLOS ONE does not copyedit accepted manuscripts, so the language in submitted articles must be clear, correct, and unambiguous. Any typographical or grammatical errors should be corrected at revision, so please note any specific errors here.

Reviewer #1: Yes

Reviewer #2: Yes

**********

5. Review Comments to the Author

Please use the space provided to explain your answers to the questions above. You may also include additional comments for the author, including concerns about dual publication, research ethics, or publication ethics. (Please upload your review as an attachment if it exceeds 20,000 characters)

Reviewer #1: Clearly state the research objective or hypothesis.

Provide a brief background on post-traumatic osteoarthritis (PTOA), its prevalence, and the need for understanding its molecular mechanisms.

Describe the methods used for inducing PTOA in the mouse models.

Provide more details on the single-cell RNA sequencing technique and data analysis methods.

Discuss the significance of using PTOA-susceptible and PTOA-resistant mouse strains in the study.

Include information on the sample size and statistical analysis performed.

Provide a comprehensive review of the relevant literature on PTOA and immune cell involvement.

Discuss the potential implications of the observed differences in monocyte and macrophage subpopulations between MRL and B6 joints.

Discuss the role of CD206+ macrophages and Trem2 receptor in PTOA development or resolution.

Consider comparing the findings with previous studies in the field.

Discuss the potential mechanisms underlying the enhanced clearance of debris and apoptotic cells in MRL injured joints.

Discuss the limitations of the study, such as the use of animal models and the extrapolation to human PTOA.

Provide a clear conclusion summarizing the key findings and their implications.

Ensure that the manuscript adheres to the journal's formatting and citation guidelines.

Include a discussion on the potential clinical relevance of the findings and their impact on PTOA treatment strategies.

Consider including visualizations or figures to illustrate the single-cell RNA sequencing results.

Discuss the potential future research directions based on the study findings.

Consider discussing the limitations and challenges of translating the findings to human PTOA.

Provide a comprehensive list of references to support the research background and contextualize the findings.

Consider the potential implications of the study for personalized medicine approaches in PTOA management

Reviewer #2: Dear Author,

Thank you for submitting your article on the use of in silico toxicity prediction and Cell Painting techniques for toxicity assessment. After careful evaluation, we regret to inform you that we are unable to accept your manuscript for publication in our journal. We have identified several concerns that need to be addressed before reconsideration:

Limited Novelty: While the use of in silico toxicity prediction and Cell Painting techniques is of interest, the presented findings do not provide significant new insights beyond what has already been reported in the literature.

Insufficient Contextualization: The introduction does not adequately establish the importance and relevance of in silico toxicity prediction and the limitations of current approaches. It is crucial to provide a comprehensive overview of the challenges in toxicology testing and the potential benefits of integrating computational methods.

Lack of Comparative Analysis: The article does not compare the findings of the study with previous studies or existing knowledge in the field. A more thorough comparison would help to assess the significance and contribution of the current study.

Incomplete Methodological Description: The article lacks a detailed description of the computational approaches used for in silico toxicity prediction and the specific parameters employed. It is important to provide sufficient information on the data integration, feature extraction, and model training processes to ensure the validity and reproducibility of the results.

Inadequate Discussion of Functional Implications: The article does not sufficiently discuss the functional implications of using a myoblast cell line for toxicity assessment and the relevance of these findings to broader toxicological endpoints. A more comprehensive discussion of the functional aspects would enhance the understanding of the findings.

Insufficient Literature Review: The article lacks a thorough review of the existing literature on in silico toxicity prediction, Cell Painting techniques, and their applications in toxicology. A comprehensive review would provide a broader context for the presented findings.

Lack of Clarity in Results Presentation: The presentation of results and findings in the article is unclear and lacks proper organization. The information provided should be presented in a logical and concise manner to facilitate understanding.

Incomplete Discussion of Limitations: The article does not adequately address the limitations of the study, such as the choice of the myoblast cell line, the representativeness of the panel of myotoxicants, or the generalizability of the findings. It is important to acknowledge and discuss these limitations to provide a balanced interpretation of the results.

**********

6. PLOS authors have the option to publish the peer review history of their article (what does this mean? ). If published, this will include your full peer review and any attached files.

If you choose “no”, your identity will remain anonymous but your review may still be made public.

**Do you want your identity to be public for this peer review?** For information about this choice, including consent withdrawal, please see our Privacy Policy .

Reviewer #1: No

Reviewer #2: No

**********

[NOTE: If reviewer comments were submitted as an attachment file, they will be attached to this email and accessible via the submission site. Please log into your account, locate the manuscript record, and check for the action link "View Attachments". If this link does not appear, there are no attachment files.]

While revising your submission, please upload your figure files to the Preflight Analysis and Conversion Engine (PACE) digital diagnostic tool, https://pacev2.apexcovantage.com/ . PACE helps ensure that figures meet PLOS requirements. To use PACE, you must first register as a user. Registration is free. Then, login and navigate to the UPLOAD tab, where you will find detailed instructions on how to use the tool. If you encounter any issues or have any questions when using PACE, please email PLOS at figures@plos.org . Please note that Supporting Information files do not need this step.

# Author comment: Author response to Decision Letter 1

**DOI:** 10.1371/journal.pone.0320040.r003

19 Aug 2024

Reviewer #1:

Unfortunately, most of the comments and suggestions from Reviewer #1 were of no help to further improving our manuscript, notably because the discussed subject is not post-traumatic osteoarthritis (PTOA), as mentioned in the reviewer’s comments. This suggests a potential misunderstanding, miscommunication regarding the scope of our work, or a mixup with another manuscript may have occurred.

Clearly state the research objective or hypothesis.

The research objective and hypothesis are thoroughly stated.

Provide a brief background on post-traumatic osteoarthritis (PTOA), its prevalence, and the need for understanding its molecular mechanisms.

Describe the methods used for inducing PTOA in the mouse models.

Discuss the significance of using PTOA-susceptible and PTOA-resistant mouse strains in the study.

Provide a comprehensive review of the relevant literature on PTOA and immune cell involvement.

This comment is not relevant in the context of our study.

Post-traumatic osteoarthritis (PTOA) is not the subject of this research.

Discuss the potential implications of the observed differences in monocyte and macrophage subpopulations between MRL and B6 joints.

No mention of monocytes, macrophage populations, MRL or B6 joints were made in the manuscript.

Discuss the role of CD206+ macrophages and Trem2 receptor in PTOA development or resolution.

Post-traumatic osteoarthritis (PTOA) is not the subject of this research.

Consider comparing the findings with previous studies in the field.

Discuss the potential mechanisms underlying the enhanced clearance of debris and apoptotic cells in MRL injured joints.

Post-traumatic osteoarthritis (PTOA) is not the subject of this research.

Discuss the limitations of the study, such as the use of animal models and the extrapolation to human PTOA.

Post-traumatic osteoarthritis (PTOA) is not the subject of this research.

Provide a clear conclusion summarizing the key findings and their implications.

A conclusion was already presented in the manuscript, but was rewritten for clarity.

Ensure that the manuscript adheres to the journal's formatting and citation guidelines.

We made sure that the manuscript strictly followed the PLoS: One formatting and citation policies.

Include a discussion on the potential clinical relevance of the findings and their impact on PTOA treatment strategies.

Post-traumatic osteoarthritis (PTOA) is not the subject of this research.

Consider including visualizations or figures to illustrate the single-cell RNA sequencing results.

No single-cell RNA sequencing was performed in this research study.

Discuss the potential future research directions based on the study findings.

Consider discussing the limitations and challenges of translating the findings to human PTOA.

Post-traumatic osteoarthritis (PTOA) is not the subject of this research.

Provide a comprehensive list of references to support the research background and contextualize the findings.

Multiple references supporting the research background and our findings are provided.

Consider the potential implications of the study for personalized medicine approaches in PTOA management

Post-traumatic osteoarthritis (PTOA) is not the subject of this research.

Reviewer #2

Limited Novelty: While the use of in silico toxicity prediction and Cell Painting techniques is of interest, the presented findings do not provide significant new insights beyond what has already been reported in the literature.

There is no data on muscle toxicity. Cell context/identity is often central to determine responses to toxicants, affected by the cell-specific expression of targets, and toxifying/de-toxifying metabolic pathways and variable levels of cellular defenses.

Moreover, in this manuscript we report the first instance of phenotypic cell profiling of C2C12 cells in the litterature. To our knowledge, Cell Painting or other kind of profiling assay has never been conducted before this study on this cell line.

Insufficient Contextualization: The introduction does not adequately establish the importance and relevance of in silico toxicity prediction and the limitations of current approaches. It is crucial to provide a comprehensive overview of the challenges in toxicology testing and the potential benefits of integrating computational methods.

The introduction has been revised to highlight the relevance and the importance of in silico prediction of toxicity in skeletal muscle cells.

Lack of Comparative Analysis: The article does not compare the findings of the study with previous studies or existing knowledge in the field. A more thorough comparison would help to assess the significance and contribution of the current study.

Due to the lack of other phenotypic profiling studies of C2C12 cells, comparative analysis could not be performed in the current state of research, and comparison with data from other cell types or cell lines is out of the scope of this study.

Incomplete Methodological Description: The article lacks a detailed description of the computational approaches used for in silico toxicity prediction and the specific parameters employed. It is important to provide sufficient information on the data integration, feature extraction, and model training processes to ensure the validity and reproducibility of the results.

All the computational approaches used in this study have all been thoroughly documented and described in the Methods section of our manuscript. If any specific methodology is unclear, we are open to provide any additional detail that is not currently present in the manuscript. However, due to the lack of specific mention in the reviewer’s comment, it is unclear for us to know which methodological approach is lacking detail.

Inadequate Discussion of Functional Implications: The article does not sufficiently discuss the functional implications of using a myoblast cell line for toxicity assessment and the relevance of these findings to broader toxicological endpoints. A more comprehensive discussion of the functional aspects would enhance the understanding of the findings.

The C2C12 cell line provides a robust, reproducible model of both myoblasts (a stem-cell like cell) involved in repair and regeneration and myotubes, a differentiated muscle cell type that expresses muscle specific genes and executes muscle specific processes not modeled in other in vitro cell painting studies. A detailed discussion on the relevance of the findings is now provided in the manuscript as suggested.

Insufficient Literature Review: The article lacks a thorough review of the existing literature on in silico toxicity prediction, Cell Painting techniques, and their applications in toxicology. A comprehensive review would provide a broader context for the presented findings.

The literature review in the introduction was greatly revised to better represent the state of research in the field.

Lack of Clarity in Results Presentation: The presentation of results and findings in the article is unclear and lacks proper organization. The information provided should be presented in a logical and concise manner to facilitate understanding.

The description of results was clarified, and additional analysis was conducted to support our claims, with for instance a study underlying the changes in myoblast nuclei size when treated with colchicine.

Incomplete Discussion of Limitations: The article does not adequately address the limitations of the study, such as the choice of the myoblast cell line, the representativeness of the panel of myotoxicants, or the generalizability of the findings. It is important to acknowledge and discuss these limitations to provide a balanced interpretation of the results.

A section dedicated to the limitations of our approach and techniques has been added. The discussion section of the manuscript was also completely reworked for more accurate language and clarity.

Attachment Submitted filename: Reply to individual comments.pdf

# Peer review: Decision Letter 1

**DOI:** 10.1371/journal.pone.0320040.r004

**Authors:** Zhiwen Luo

30 Oct 2024

PONE-D-24-09028R1Drug-induced cytotoxicity prediction in muscle cells, an application of the Cell Painting assayPLOS ONE

Dear Dr. Lambert,

Thank you for submitting your manuscript to PLOS ONE. After careful consideration, we feel that it has merit but does not fully meet PLOS ONE’s publication criteria as it currently stands. Therefore, we invite you to submit a revised version of the manuscript that addresses the points raised during the review process.

Please submit your revised manuscript by Dec 14 2024 11:59PM. If you will need more time than this to complete your revisions, please reply to this message or contact the journal office at plosone@plos.org . When you're ready to submit your revision, log on to https://www.editorialmanager.com/pone/ and select the 'Submissions Needing Revision' folder to locate your manuscript file.

Please include the following items when submitting your revised manuscript:

- A rebuttal letter that responds to each point raised by the academic editor and reviewer(s). You should upload this letter as a separate file labeled 'Response to Reviewers'.
- A marked-up copy of your manuscript that highlights changes made to the original version. You should upload this as a separate file labeled 'Revised Manuscript with Track Changes'.
- An unmarked version of your revised paper without tracked changes. You should upload this as a separate file labeled 'Manuscript'.

If you would like to make changes to your financial disclosure, please include your updated statement in your cover letter. Guidelines for resubmitting your figure files are available below the reviewer comments at the end of this letter.

If applicable, we recommend that you deposit your laboratory protocols in protocols.io to enhance the reproducibility of your results. Protocols.io assigns your protocol its own identifier (DOI) so that it can be cited independently in the future. For instructions see: https://journals.plos.org/plosone/s/submission-guidelines#loc-laboratory-protocols . Additionally, PLOS ONE offers an option for publishing peer-reviewed Lab Protocol articles, which describe protocols hosted on protocols.io. Read more information on sharing protocols at https://plos.org/protocols?utm_medium=editorial-email&utm_source=authorletters&utm_campaign=protocols .

We look forward to receiving your revised manuscript.

Kind regards,

Zhiwen Luo

Academic Editor

PLOS ONE

**Additional Editor Comments:**

Thank you for submitting your manuscript to the Journal and as voucan see that the reviewer think your manuscript is interesting and provide valuable comments for your reference. Please submit the revised manuscript ASAP and also include a rebuttal that would clearly list all the responses to the reviewer's comments.

[Note: HTML markup is below. Please do not edit.]

Reviewers' comments:

Reviewer's Responses to Questions

**Comments to the Author**

1. If the authors have adequately addressed your comments raised in a previous round of review and you feel that this manuscript is now acceptable for publication, you may indicate that here to bypass the “Comments to the Author” section, enter your conflict of interest statement in the “Confidential to Editor” section, and submit your "Accept" recommendation.

Reviewer #3: All comments have been addressed

Reviewer #4: All comments have been addressed

Reviewer #5: All comments have been addressed

Reviewer #6: (No Response)

**********

2. Is the manuscript technically sound, and do the data support the conclusions?

The manuscript must describe a technically sound piece of scientific research with data that supports the conclusions. Experiments must have been conducted rigorously, with appropriate controls, replication, and sample sizes. The conclusions must be drawn appropriately based on the data presented. 

Reviewer #3: Yes

Reviewer #4: Yes

Reviewer #5: Yes

Reviewer #6: Partly

**********

3. Has the statistical analysis been performed appropriately and rigorously? 

Reviewer #3: I Don't Know

Reviewer #4: Yes

Reviewer #5: Yes

Reviewer #6: I Don't Know

**********

4. Have the authors made all data underlying the findings in their manuscript fully available?

The PLOS Data policy requires authors to make all data underlying the findings described in their manuscript fully available without restriction, with rare exception (please refer to the Data Availability Statement in the manuscript PDF file). The data should be provided as part of the manuscript or its supporting information, or deposited to a public repository. For example, in addition to summary statistics, the data points behind means, medians and variance measures should be available. If there are restrictions on publicly sharing data—e.g. participant privacy or use of data from a third party—those must be specified.

Reviewer #3: Yes

Reviewer #4: Yes

Reviewer #5: Yes

Reviewer #6: No

**********

5. Is the manuscript presented in an intelligible fashion and written in standard English?

PLOS ONE does not copyedit accepted manuscripts, so the language in submitted articles must be clear, correct, and unambiguous. Any typographical or grammatical errors should be corrected at revision, so please note any specific errors here.

Reviewer #3: Yes

Reviewer #4: Yes

Reviewer #5: Yes

Reviewer #6: Yes

**********

6. Review Comments to the Author

Please use the space provided to explain your answers to the questions above. You may also include additional comments for the author, including concerns about dual publication, research ethics, or publication ethics. (Please upload your review as an attachment if it exceeds 20,000 characters)

Reviewer #3: The revised manuscript is significantly improved but there are some issues with the formatting and content.

Lines 130-134 and 230-234 seem to be the same but the references are different.

C2C12 cell line introduction section should be placed in the in Introduction chapter.

References are missing in line 83, 84 and 89.

Page 10 of the revised manuscript, lines 237 and 239: there are no Figure 2A, 2C, and 2D. I guess the corresponding sentences refer to Figure 1. Figure 1B is not cited anywhere in the manuscript.

Lines 463 and 468 in the discussion, what number does the "xxx" correspond to?

Reviewer #4: I read with interest the current manuscript and as all comment addressed, i recommend it is worth to publish now.

Reviewer #5: Nothing much. The revised paper and images (may be, change the graph colors and designs - the excel designs are too obvious - just a personal recommendation) have enough clarity.

All the sections have been revised thoroughly.

Reviewer #6: The authors have presented an application of the cell painting assay to evaluate drug-induced mytotoxicity in C2C12 cells, both as myoblasts and having undergone differentiation to . While the application of the technique is well intended in the field of toxicology, there are several concerns regarding the methodology, particularly the machine learning and statistical analysis.

In a nutshell, the sources stated in the data availability statement (Zenodo and GitHub) are not accessible and therefore cannot be evaluated during peer-review. This is a very strong ground for rejection, but as a courtesy I am still willing to review the shared material and give the authors a chance to share the remaining data at the time of revision. Please find below the concerns that need to be addressed in order to prepare the submission for publication:

1. In Figure 1, please include the colour mapping in the figure itself. The images in the figure also lack a scale. Please justify your reason to highlight the 100 mM exposure of colchicine in the manuscript text associated with the figure.

2. Please prepare a supplementary table/spreadsheet with all the identified features following analysis in Harmony and CellProfiler (lines 114-115).

3. Please make available the image analysis pipeline for Harmony and CellProfiler as supplementary data (say on GitHub).

4. In line 121, how were the optimal phenotypes selected (what criteria did you use for this)? This requires further elaboration. Where can I find the output for the analysis that was used to make this selection?

5. Please include significance values (adjusted p values, R2 values etc.) wherever you have claimed significance in the results section text of the manuscript. The reader must not always have to look at the linked figure/supplementary data to find this.

6. In Figure 2, can the cell count values also be plotted as percentage of control like the viability dataset? This will make it easier than comparing absolute to normalised median values in the same plot. Please also add significance symbols for at least the comparison with the control for each readout.

7. In line 138, you mention the ATP based assay for cell cytotoxicity. It would be nice to discuss why you didn’t go for other more well-established assays in toxicology, such as the Alamar blue or MTS assays. Do any of the included drugs in the panel have an impact on energy/ATP metabolism, especially glycolysis which is more-prominent in-vitro?

8. In line 152 – 154, I cannot locate the dataset for the results that have been described. You highlight certain phenotypic changes here, but where are the supporting images?

9. In Figures 3 and 4, please include the actual R2 value with each plotted bar. Also improve the readability of the y axis labels with the plotted bars (you can add axis delimiters).

10. For the random forest machine learning, have you calculated the AUC of ROC, sensitivity, specificity and accuracy for each model? I cannot find the code used to generate the models as well.

11. Please identify the top features of the dataset that contribute to the prediction models, particularly to the combined model. For example, this can be done by calculating SHAP scores (https://doi.org/10.1038/s42256-019-0138-9) that can identify important features from the random forest models that were generated in the study.

12. I would recommend to move Figure 5 to the supplementary and instead generate a new figure from the same dataset that clearly visualises the top 30 – 50 features. The current figure is good at demonstrating the clustering of the drug responses, but not much more beyond that. The top features should be then discussed as well.

13. In lines 190 – 191, you state that there is no correlation between cell count, viability and the resulting clusters. Please mention which results you are referring to when making this statement as it is unclear.

14. Please prepare a supplementary table/spreadsheet with all features that were identified in the random forest machine learning analysis, for individual as well as combined models.

15. Have you performed any exploratory analysis with PCA/OPLS-DA to identify important features that have an impact on the dataset? Have you compared this and other regression models with the random forest machine learning?

16. Please justify your choice of random forest machine learning in the discussion. Have other models been previously applied to cell painting assays? How does your model perform compared to other studies (independent of cell type, only concerning cell painting to predict features).

17. I cannot locate the data on the basis of which the results in lines 214 – 218 have been described. This needs to be properly tagged to the corresponding dataset and further elaborated upon.

18. I am uncertain with the statement in line 228. Please include the analysis in the supplementary data without the selected 20% induction threshold (please clarify in lines 224 – 225 how you arrived at this) to support this statement and/or rewrite in a more factual manner.

19. You have mentioned Figure 6 in the results (line 223). Please describe in the text what you interpret from the included figure.

20. It is unclear where the features highlighted in Table 2 come from in terms of the applied machine learning (from only Harmony, only CellProfiler or both). Please clarify in the text.

21. In line 233, are you referring to Figure S3 instead of S4? Please check.

22. In line 237, you mention that you calculated Euclidean distances as well, but with “poor results”. Please provide these results in the supplementary and indicate the nature of the results in more detail.

23. Supplementary Table 2 has not been attributed in the text of the results section (paragraph from line 247).

24. Please indicate the results from which the statement in line 260 has been mentioned.

25. In line 325, do you mean to state Figure S4 instead of S3? Please check.

26. The limitations section in the discussion is introducing additional results that are not in the results section of the manuscript (lines 320 – 325). This should be moved to the results section and restructured appropriately. Additionally, provide information about this analysis in the methodology as well.

27. In the methodology section, please provide more information of the drug library. What was the inclusion/exclusion criteria? State the reasoning behind the choice of the drug library.

28. The cell painting protocol needs to be further elaborated in the methodology section as it is a key method of the paper. Simply citing previous work with the application is not sufficient. Also, include a detailed list of reagents used for the staining as a table in the supplementary data. In general, it is recommended to provide catalogue numbers along with the supplier details for each reagent and version number for each tool in the methodology section.

29. In the methodology section, the data analysis part requires further elaboration. State the different statistical tests used, post-hoc analysis conducted, significance thresholds and other relevant information. Do this for each analysis that has been included for the manuscript.

30. There is also a need to further elaborate on the model building in the methodology section. How many trees were generated, how many iterations were made, what were the significance thresholds, and were there any corrections for multiple testing made?

**********

7. PLOS authors have the option to publish the peer review history of their article (what does this mean? ). If published, this will include your full peer review and any attached files.

If you choose “no”, your identity will remain anonymous but your review may still be made public.

**Do you want your identity to be public for this peer review?** For information about this choice, including consent withdrawal, please see our Privacy Policy .

Reviewer #3: **Yes: ** Zsolt Sarang

Reviewer #4: **Yes: ** Mohammad Amrollahi-Sharifabadi, PhD in Toxicology

Reviewer #5: No

Reviewer #6: No

**********

[NOTE: If reviewer comments were submitted as an attachment file, they will be attached to this email and accessible via the submission site. Please log into your account, locate the manuscript record, and check for the action link "View Attachments". If this link does not appear, there are no attachment files.]

While revising your submission, please upload your figure files to the Preflight Analysis and Conversion Engine (PACE) digital diagnostic tool, https://pacev2.apexcovantage.com/ . PACE helps ensure that figures meet PLOS requirements. To use PACE, you must first register as a user. Registration is free. Then, login and navigate to the UPLOAD tab, where you will find detailed instructions on how to use the tool. If you encounter any issues or have any questions when using PACE, please email PLOS at figures@plos.org . Please note that Supporting Information files do not need this step.

# Author comment: Author response to Decision Letter 2

**DOI:** 10.1371/journal.pone.0320040.r005

14 Dec 2024

Review Comments to the Author

Reviewer #3: The revised manuscript is significantly improved but there are some issues with the formatting and content.

Lines 130-134 and 230-234 seem to be the same but the references are different.

C2C12 cell line introduction section should be placed in the in Introduction chapter.

We thank the reviewer for this useful comment. The introduction of the C2C12 cell line has been moved to the Introduction section as suggested. The mismatch of references was due to a mixup between the tracked changes version and the final manuscript version, which has been corrected.

References are missing in line 83, 84 and 89.

We thank the reviewer for catching this reference issue. A mixup between the tracked changes version and the final manuscript version was identified, and this mistake has been corrected in this new resubmission.

Page 10 of the revised manuscript, lines 237 and 239: there are no Figure 2A, 2C, and 2D. I guess the corresponding sentences refer to Figure 1. Figure 1B is not cited anywhere in the manuscript.

Figure 2 has been renamed in the final manuscript, and the identified issue was due to the same mixup of the tracked changes version, which has been corrected.

Lines 463 and 468 in the discussion, what number does the "xxx" correspond to?

We want to thank the reviewer for pointing out that mistake. This figure reference placeholder was still present in the tracked changes version of the previous submission due to a mixup in versioning. This has not been corrected. We apologize for the inconvenience.

Reviewer #5: Nothing much. The revised paper and images (may be, change the graph colors and designs - the excel designs are too obvious - just a personal recommendation) have enough clarity.

All the sections have been revised thoroughly.

We thank the reviewer for their positive feedback and helpful suggestion regarding the graph design. We have revised our figures using a more professional visualization approach, moving away from Excel styling to enhance the clarity and professional appearance of our results, now using Python.

Reviewer #6: The authors have presented an application of the cell painting assay to evaluate drug-induced mytotoxicity in C2C12 cells, both as myoblasts and having undergone differentiation to . While the application of the technique is well intended in the field of toxicology, there are several concerns regarding the methodology, particularly the machine learning and statistical analysis.

In a nutshell, the sources stated in the data availability statement (Zenodo and GitHub) are not accessible and therefore cannot be evaluated during peer-review. This is a very strong ground for rejection, but as a courtesy I am still willing to review the shared material and give the authors a chance to share the remaining data at the time of revision. Please find below the concerns that need to be addressed in order to prepare the submission for publication:

1. In Figure 1, please include the colour mapping in the figure itself. The images in the figure also lack a scale. Please justify your reason to highlight the 100 mM exposure of colchicine in the manuscript text associated with the figure.

We thank the reviewer for this comment. Color coding of the different image channels has been added to Figure 1, as well as a scale to help the representation of object size. COLC 100nM was selected as it is the lowest concentration where a significant decrease in cell count was observed. An explanation has been added to the manuscript text.

2. Please prepare a supplementary table/spreadsheet with all the identified features following analysis in Harmony and CellProfiler (lines 114-115).

The supplementary table (Table S1) was included to summarize the different types of features extracted with the two feature extraction pipelines, obtained with Harmony and Cell Profiler. Unfortunately, due to the extensive number of features calculated with each feature extraction pipeline, we are unable to include an exhaustive list of features. With this solution, we hope the table summary will help the understanding of the different types of readouts studied in this manuscript.

3. Please make available the image analysis pipeline for Harmony and CellProfiler as supplementary data (say on GitHub).

We thank the reviewer for this important suggestion. The Cell Profiler pipelines, the Jupyter notebooks as well as the Python scripts used for the analysis have now been made available on the GitHub repository and are freely accessible. Due to the close source nature of the Harmony building blocks pipeline, we are not able to share it as is and would require more expertise to extract it from Harmony. We will however make it available on GitHub as soon as we are able to. We are sorry for this inconvenience.

4. In line 121, how were the optimal phenotypes selected (what criteria did you use for this)? This requires further elaboration. Where can I find the output for the analysis that was used to make this selection?

Unfortunately no in-depth study was conducted for selecting the Robust MAD profiles, but a qualitative analysis on the heatmaps of the different profiles, as well as compound separation on several PCAs showed that profiles obtained with Zscore or MinMax normalization were more subject to inter-plate variability compared to the ones obtained with the RobustMAD method.

5. Please include significance values (adjusted p values, R2 values etc.) wherever you have claimed significance in the results section text of the manuscript. The reader must not always have to look at the linked figure/supplementary data to find this.

The word "significant" was sometimes wrongly used in the context of the manuscript, where no significance tests were actually conducted. We have rephrased our claims to reflect this information, and we have conducted statistical tests when possible. Please accept our apologies, as English is not the mother tongue of all the authors. Additionally, cell count data has now been normalized to the vehicle controls to allow easier comparison to cell viability data. Statistical tests have been conducted for cell viability and cell count data. For each concentration, significance has been tested against the vehicle controls and symbols were added on top of each bar to indicate the significance level.

6. In Figure 2, can the cell count values also be plotted as percentage of control like the viability dataset? This will make it easier than comparing absolute to normalised median values in the same plot. Please also add significance symbols for at least the comparison with the control for each readout.

We thank the reviewer for this important comment. Consequently, cell count data has been normalized to the vehicle controls to allow easier comparison to cell viability data, and better readability of the figure. Statistical tests have also been conducted for each concentration against the vehicle controls to assess statistical significance, and symbols were added on top of each bar. We also reverted our figure to a barplot format to make the quantity of information easier to read.

7. In line 138, you mention the ATP based assay for cell cytotoxicity. It would be nice to discuss why you didn’t go for other more well-established assays in toxicology, such as the Alamar blue or MTS assays. Do any of the included drugs in the panel have an impact on energy/ATP metabolism, especially glycolysis which is more-prominent in-vitro?

Thank you for this insightful question about our choice of cell viability assay. Our choice quickly ported to CellTiter-Glo, as it is known that ATP measurement provides a rapid and sensitive indicator of metabolically active cells. Existing studies are showing its ability to detect subtle changes in cell health before morphological changes become apparent (Crouch et al., 1993, DOI 10.1016/0022-1759(93)90011-u ). While Alamar Blue and MTS assays are indeed well-established, ATP assays often demonstrate higher sensitivity, particularly when assessing early cytotoxic effects. We indeed suspect that some of our drugs may impact ATP metabolism, especially in the mitochondria, which is another motivation to use an ATP-based cell viability assay in this study to capture these effects.

8. In line 152 – 154, I cannot locate the dataset for the results that have been described. You highlight certain phenotypic changes here, but where are the supporting images?

A reference to Figure 1 was missing from the manuscript text, and was hereby added thanks to this suggestion.

9. In Figures 3 and 4, please include the actual R2 value with each plotted bar. Also improve the readability of the y axis labels with the plotted bars (you can add axis delimiters).

We thank the reviewer for this suggestion which will allow a better readability of our figure. All R2 values have been added to each bar plotted in this figure.

10. For the random forest machine learning, have you calculated the AUC of ROC, sensitivity, specificity and accuracy for each model? I cannot find the code used to generate the models as well.

While ROC AUC, sensitivity and specificity are indeed crucial metrics that must be monitored when building classification models, they cannot be computed when building regression models, such as in this study. That is why we chose to assess the performance of the regression models by means of the R-squared, and the MSE, not shown in the results as they resulted in equal model rankings. The code used to generate the global and individual models has been submitted to the GitHub repository.

11. Please identify the top features of the dataset that contribute to the prediction models, particularly to the combined model. For example, this can be done by calculating SHAP scores (https://doi.org/10.1038/s42256-019-0138-9) that can identify important features from the random forest models that were generated in the study.

We want to thank the reviewer for this suggestion regarding SHAP analysis. SHAP values are robust and well-known indicators for the contribution of each feature to ML models. However in the present study, feature contribution for each model has already been assessed via computation of the feature importance extracted during model building. Our primary goal with this approach was to assess the global importance of features across the entire dataset, which is what RF feature importance already provides. While SHAP values can provide an additional perspective through local interpretability of the features, we consider that our current analysis already satisfies the research questions and identifies the key phenotypic properties associated with cell viability.

12. I would recommend to move Figure 5 to the supplementary and instead generate a new figure from the same dataset that clearly visualises the top 30 – 50 features. The current figure is good at demonstrating the clustering of the drug responses, but not much more beyond that. The top features should be then discussed as well.

We want to thank the reviewer for this comment. Accordingly, the original figure has been moved to supplementary data, and a new Figure using the same dataset has been replaced as Figure 5, where the top 30 features according to their importance in individual models are analyzed and clustered.

13. In lines 190 – 191, you state that there is no correlation between cell count, viability and the resulting clusters. Please mention which results you are referring to when making this statement as it is unclear.

This observation taken from the original Figure 5 were an attempt at proving that important features in individual compound models and the significant decrease in count or viability were not correlated. This part of the manuscript is not relevant anymore with the change in Figure 5, and has been discarded.

14. Please prepare a supplementary table/spreadsheet with all features that were identified in the random forest machine learning analysis, for individual as well as combined models.

Due to the important size of the tables containing the feature importance of each model, we have added the table of Random Forest feature importances computed for the global model built with Harmony features in supplementary materials. All features with an importance above 0.001 are displayed in this table, which make up 71 out of 310 total Harmony features.

15. Have you performed any exploratory analysis with PCA/OPLS-DA to identify important features that have an impact on the dataset? Have you compared this and other regression models with the random forest machine learning?

Multiple linear regression, polynomial regression and simpler decision trees were also tested in preliminary studies, but Random Forests yielded the best results in this use case. Several PCAs have been conducted for visualization purposes, but the contribution of each phenotypic property to the principal components have not been studied here. This idea could be of interest for such models in future experiments, but we are confident that the RF feature importance used in the present study is a reliable metric to assess the impact of features on the predictive models.

16. Please justify your choice of random forest machine learning in the discussion. Have other models been previously applied to cell painting assays? How does your model perform compared to other studies (independent of cell type, only concerning cell painting to predict features).

Random Forests was chosen after a comparison to linear and polynomial regression models, which didn’t give any satisfactory results. To our knowledge, this study is the first occurrence of a Machine Learning model leveraging Cell Painting features to predict cell viability.

17. I cannot locate the data on the basis of which the results in lines 214 – 218 have been described. This needs to be properly tagged to the corresponding dataset and further elaborated upon.

The model performance for C2C12 myotubes results are given in Figures 3 and 4. A reference to these figures has been added to the manuscript to improve the clarity of this paragraph.

18. I am uncertain with the statement in line 228. Please include the analysis in the supplementary data without the selected 20% induction threshold (please clarify in lines 224 – 225 how you arrived at this) to support this statement and/or rewrite in a more factual manner.

The corresponding text was rephrased for clarity, and a reference to previous works was added. The 20% induction threshold was chosen empirically and also motivated by previous works, such as in Schneidewind et al 2020 (DOI 10.1002/cbic.202000381), in which phenotypes are filtered for induction values between 17% and 37%. We hence believe that our 20% lower threshold is a good compromise between filtering out noisy phenotypes, and maintaining a good signal from the meaningful ones.

19. You have mentioned Figure 6 in the results (line 223). Please describe in the text what you interpret from the included figure.

The interpretation of the results displayed in Figure 6 has been consolidated, and were indeed lacking depth in the previous version of the manuscript. We want to thank the reviewer for pointing out this omission.

20. It is unclear where the features highlighted in Table 2 come from in terms of the applied machine learning (from only Harmony, only CellProfiler or both). Please clarify in the text.

Clarification on the origin of the dataset has been added to the text and to the table legend. Harmony data was used to produce these results.

21. In line 233, are you referring to Figure S3 instead of S4? Please check.

We are grateful to the reviewer for catching this typo. The text was indeed referring to Figure S3, and not Figure S4. This reference has been corrected in the manuscript.

22. In line 237, you mention that you calculated Euclidean distances as well, but with “poor results”. Please provide these results in the supplementary and indicate the nature of the results in more detail.

The section dedicated to the Euclidean distance-based hierarchical clustering has been rephrased to reflect the exclusion criteria, and the Euclidean based figure was added as a supplementary Figure (S6).

23. Supplementar

Attachment Submitted filename: ROUND2 - ReplyToComments.pdf

# Peer review: Decision Letter 2

**DOI:** 10.1371/journal.pone.0320040.r006

**Authors:** Zhiwen Luo

9 Jan 2025

PONE-D-24-09028R2Drug-induced cytotoxicity prediction in muscle cells, an application of the Cell Painting assayPLOS ONE

Dear Dr. Lambert,

Thank you for submitting your manuscript to PLOS ONE. After careful consideration, we feel that it has merit but does not fully meet PLOS ONE’s publication criteria as it currently stands. Therefore, we invite you to submit a revised version of the manuscript that addresses the points raised during the review process.

==============================

**ACADEMIC EDITOR: ** Thank you for submitting your manuscript to the Journal and as voucan see that the reviewer think your manuscript is interesting and provide valuable comments for your reference. Please submit the revised manuscript ASAP and also include a rebuttal that would clearly list all the responses to the reviewer's comments.

==============================

Please submit your revised manuscript by Feb 23 2025 11:59PM. If you will need more time than this to complete your revisions, please reply to this message or contact the journal office at plosone@plos.org . When you're ready to submit your revision, log on to https://www.editorialmanager.com/pone/ and select the 'Submissions Needing Revision' folder to locate your manuscript file.

Please include the following items when submitting your revised manuscript:

- A rebuttal letter that responds to each point raised by the academic editor and reviewer(s). You should upload this letter as a separate file labeled 'Response to Reviewers'.
- A marked-up copy of your manuscript that highlights changes made to the original version. You should upload this as a separate file labeled 'Revised Manuscript with Track Changes'.
- An unmarked version of your revised paper without tracked changes. You should upload this as a separate file labeled 'Manuscript'.

If you would like to make changes to your financial disclosure, please include your updated statement in your cover letter. Guidelines for resubmitting your figure files are available below the reviewer comments at the end of this letter.

If applicable, we recommend that you deposit your laboratory protocols in protocols.io to enhance the reproducibility of your results. Protocols.io assigns your protocol its own identifier (DOI) so that it can be cited independently in the future. For instructions see: https://journals.plos.org/plosone/s/submission-guidelines#loc-laboratory-protocols . Additionally, PLOS ONE offers an option for publishing peer-reviewed Lab Protocol articles, which describe protocols hosted on protocols.io. Read more information on sharing protocols at https://plos.org/protocols?utm_medium=editorial-email&utm_source=authorletters&utm_campaign=protocols .

We look forward to receiving your revised manuscript.

Kind regards,

Zhiwen Luo

Academic Editor

PLOS ONE

Journal Requirements:

Please review your reference list to ensure that it is complete and correct. If you have cited papers that have been retracted, please include the rationale for doing so in the manuscript text, or remove these references and replace them with relevant current references. Any changes to the reference list should be mentioned in the rebuttal letter that accompanies your revised manuscript. If you need to cite a retracted article, indicate the article’s retracted status in the References list and also include a citation and full reference for the retraction notice.

**Additional Editor Comments:**

Thank you for submitting your manuscript to the Journal and as voucan see that the reviewer think your manuscript is interesting and provide valuable comments for your reference. Please submit the revised manuscript ASAP and also include a rebuttal that would clearly list all the responses to the reviewer's comments.

[Note: HTML markup is below. Please do not edit.]

Reviewers' comments:

Reviewer's Responses to Questions

**Comments to the Author**

1. If the authors have adequately addressed your comments raised in a previous round of review and you feel that this manuscript is now acceptable for publication, you may indicate that here to bypass the “Comments to the Author” section, enter your conflict of interest statement in the “Confidential to Editor” section, and submit your "Accept" recommendation.

Reviewer #3: All comments have been addressed

Reviewer #6: (No Response)

**********

2. Is the manuscript technically sound, and do the data support the conclusions?

The manuscript must describe a technically sound piece of scientific research with data that supports the conclusions. Experiments must have been conducted rigorously, with appropriate controls, replication, and sample sizes. The conclusions must be drawn appropriately based on the data presented. 

Reviewer #3: Yes

Reviewer #6: Yes

**********

3. Has the statistical analysis been performed appropriately and rigorously? 

Reviewer #3: Yes

Reviewer #6: Yes

**********

4. Have the authors made all data underlying the findings in their manuscript fully available?

The PLOS Data policy requires authors to make all data underlying the findings described in their manuscript fully available without restriction, with rare exception (please refer to the Data Availability Statement in the manuscript PDF file). The data should be provided as part of the manuscript or its supporting information, or deposited to a public repository. For example, in addition to summary statistics, the data points behind means, medians and variance measures should be available. If there are restrictions on publicly sharing data—e.g. participant privacy or use of data from a third party—those must be specified.

Reviewer #3: Yes

Reviewer #6: Yes

**********

5. Is the manuscript presented in an intelligible fashion and written in standard English?

PLOS ONE does not copyedit accepted manuscripts, so the language in submitted articles must be clear, correct, and unambiguous. Any typographical or grammatical errors should be corrected at revision, so please note any specific errors here.

Reviewer #3: Yes

Reviewer #6: Yes

**********

6. Review Comments to the Author

Please use the space provided to explain your answers to the questions above. You may also include additional comments for the author, including concerns about dual publication, research ethics, or publication ethics. (Please upload your review as an attachment if it exceeds 20,000 characters)

Reviewer #3: My comments and questions were addressed in a satisfactory way. I have no further questions to the authors and recommend the acceptance of the manuscript.

Reviewer #6: I would like to thank the authors for addressing the raised concerns in the previous round of review. I am satisfied with the responses provided by the authors and appreciate the improvements in the manuscript. I now only have some minor recommendations, after which I will deem the manuscript acceptable for publication:

1. In Figure 1, please increase the font size of the scale bar label and mention this in the figure legend as well.

2. In Figure 2, the significance symbols are overlapping with the error bars in some of the plots, making it difficult to interpret. Please correct the placement of these and include what they indicate in the figure legend.

3. Please use a uniform style to refer to the figures in the manuscript text (such as Fig 1 or Figure 1). Please also name the supplementary figures/tables consistently in the text and supporting information document (such as Figure S1 or Supplementary Figure 1).

4. In the supporting information document, I recommend placing each supplementary table/figure on a separate page, so that there are no cropping issues (check Supplementary Figure 3) or issues with following the table/figure legends.

5. I cannot locate the LC50 information in the supporting data as stated in line 132. Please look into this and update as required.

**********

7. PLOS authors have the option to publish the peer review history of their article (what does this mean? ). If published, this will include your full peer review and any attached files.

If you choose “no”, your identity will remain anonymous but your review may still be made public.

**Do you want your identity to be public for this peer review?** For information about this choice, including consent withdrawal, please see our Privacy Policy .

Reviewer #3: No

Reviewer #6: No

**********

[NOTE: If reviewer comments were submitted as an attachment file, they will be attached to this email and accessible via the submission site. Please log into your account, locate the manuscript record, and check for the action link "View Attachments". If this link does not appear, there are no attachment files.]

While revising your submission, please upload your figure files to the Preflight Analysis and Conversion Engine (PACE) digital diagnostic tool, https://pacev2.apexcovantage.com/ . PACE helps ensure that figures meet PLOS requirements. To use PACE, you must first register as a user. Registration is free. Then, login and navigate to the UPLOAD tab, where you will find detailed instructions on how to use the tool. If you encounter any issues or have any questions when using PACE, please email PLOS at figures@plos.org . Please note that Supporting Information files do not need this step.

# Author comment: Author response to Decision Letter 3

**DOI:** 10.1371/journal.pone.0320040.r007

1 Feb 2025

Reply to the reviewers comments

Reviewer #3: My comments and questions were addressed in a satisfactory way. I have no further questions to the authors and recommend the acceptance of the manuscript.

Reviewer #6: I would like to thank the authors for addressing the raised concerns in the previous round of review. I am satisfied with the responses provided by the authors and appreciate the improvements in the manuscript. I now only have some minor recommendations, after which I will deem the manuscript acceptable for publication:

1. In Figure 1, please increase the font size of the scale bar label and mention this in the figure legend as well.

We want to thank the reviewer for this feedback. The font size of the scale label has been significantly increased, which improves figure readability, and a mention to the caption has been added.

2. In Figure 2, the significance symbols are overlapping with the error bars in some of the plots, making it difficult to interpret. Please correct the placement of these and include what they indicate in the figure legend.

We thank the reviewer for catching this inconsistency in the symbol placement, which hindered the legibility of the figure. This overlap has been now corrected, and the meaning of the symbols has been explained in the caption.

3. Please use a uniform style to refer to the figures in the manuscript text (such as Fig 1 or Figure 1). Please also name the supplementary figures/tables consistently in the text and supporting information document (such as Figure S1 or Supplementary Figure 1).

The figure and table naming conventions have now been harmonized in the manuscript text and in the supplementary information document.

4. In the supporting information document, I recommend placing each supplementary table/figure on a separate page, so that there are no cropping issues (check Supplementary Figure 3) or issues with following the table/figure legends.

We want to thank the reviewer for this comment improving the legibility of the supplementary data. This improvement has been implemented and page breaks have been added for improved readability.

5. I cannot locate the LC50 information in the supporting data as stated in line 132. Please look into this and update as required.

We want to thank the reviewer for catching this oversight in the supplementary data. This has now been corrected, and the LC50 information is now available in S8 Table.

Attachment Submitted filename: ROUND3 - ReplyToComments.pdf

# Peer review: Decision Letter 3

**DOI:** 10.1371/journal.pone.0320040.r008

**Authors:** Zhiwen Luo

13 Feb 2025

Drug-induced cytotoxicity prediction in muscle cells, an application of the Cell Painting assay

PONE-D-24-09028R3

Dear Dr. Lambert,

We’re pleased to inform you that your manuscript has been judged scientifically suitable for publication and will be formally accepted for publication once it meets all outstanding technical requirements.

Within one week, you’ll receive an e-mail detailing the required amendments. When these have been addressed, you’ll receive a formal acceptance letter and your manuscript will be scheduled for publication.

An invoice will be generated when your article is formally accepted. Please note, if your institution has a publishing partnership with PLOS and your article meets the relevant criteria, all or part of your publication costs will be covered. Please make sure your user information is up-to-date by logging into Editorial Manager at Editorial Manager®  and clicking the ‘Update My Information' link at the top of the page. If you have any questions relating to publication charges, please contact our Author Billing department directly at authorbilling@plos.org.

If your institution or institutions have a press office, please notify them about your upcoming paper to help maximize its impact. If they’ll be preparing press materials, please inform our press team as soon as possible -- no later than 48 hours after receiving the formal acceptance. Your manuscript will remain under strict press embargo until 2 pm Eastern Time on the date of publication. For more information, please contact onepress@plos.org.

Kind regards,

Zhiwen Luo

Academic Editor

PLOS ONE

Additional Editor Comments (optional):

Reviewers' comments:

Reviewer's Responses to Questions

**Comments to the Author**

1. If the authors have adequately addressed your comments raised in a previous round of review and you feel that this manuscript is now acceptable for publication, you may indicate that here to bypass the “Comments to the Author” section, enter your conflict of interest statement in the “Confidential to Editor” section, and submit your "Accept" recommendation.

Reviewer #3: All comments have been addressed

Reviewer #6: All comments have been addressed

**********

2. Is the manuscript technically sound, and do the data support the conclusions?

The manuscript must describe a technically sound piece of scientific research with data that supports the conclusions. Experiments must have been conducted rigorously, with appropriate controls, replication, and sample sizes. The conclusions must be drawn appropriately based on the data presented. 

Reviewer #3: Yes

Reviewer #6: Yes

**********

3. Has the statistical analysis been performed appropriately and rigorously? 

Reviewer #3: Yes

Reviewer #6: Yes

**********

4. Have the authors made all data underlying the findings in their manuscript fully available?

The PLOS Data policy requires authors to make all data underlying the findings described in their manuscript fully available without restriction, with rare exception (please refer to the Data Availability Statement in the manuscript PDF file). The data should be provided as part of the manuscript or its supporting information, or deposited to a public repository. For example, in addition to summary statistics, the data points behind means, medians and variance measures should be available. If there are restrictions on publicly sharing data—e.g. participant privacy or use of data from a third party—those must be specified.

Reviewer #3: Yes

Reviewer #6: Yes

**********

5. Is the manuscript presented in an intelligible fashion and written in standard English?

PLOS ONE does not copyedit accepted manuscripts, so the language in submitted articles must be clear, correct, and unambiguous. Any typographical or grammatical errors should be corrected at revision, so please note any specific errors here.

Reviewer #3: Yes

Reviewer #6: Yes

**********

6. Review Comments to the Author

Please use the space provided to explain your answers to the questions above. You may also include additional comments for the author, including concerns about dual publication, research ethics, or publication ethics. (Please upload your review as an attachment if it exceeds 20,000 characters)

Reviewer #3: (No Response)

Reviewer #6: I am satisfied with the responses provided by the authors and now find the manuscript acceptable for publication. All the best!

**********

7. PLOS authors have the option to publish the peer review history of their article (what does this mean? ). If published, this will include your full peer review and any attached files.

If you choose “no”, your identity will remain anonymous but your review may still be made public.

**Do you want your identity to be public for this peer review?** For information about this choice, including consent withdrawal, please see our Privacy Policy .

Reviewer #3: **Yes: ** Zsolt Sarang

Reviewer #6: No

**********

# Editor report: Acceptance letter

**DOI:** 10.1371/journal.pone.0320040.r009

**Authors:** Zhiwen Luo

PONE-D-24-09028R3

PLOS ONE

Dear Dr. Lambert,

I'm pleased to inform you that your manuscript has been deemed suitable for publication in PLOS ONE. Congratulations! Your manuscript is now being handed over to our production team.

At this stage, our production department will prepare your paper for publication. This includes ensuring the following:

* All references, tables, and figures are properly cited

* All relevant supporting information is included in the manuscript submission,

* There are no issues that prevent the paper from being properly typeset

If revisions are needed, the production department will contact you directly to resolve them. If no revisions are needed, you will receive an email when the publication date has been set. At this time, we do not offer pre-publication proofs to authors during production of the accepted work. Please keep in mind that we are working through a large volume of accepted articles, so please give us a few weeks to review your paper and let you know the next and final steps.

Lastly, if your institution or institutions have a press office, please let them know about your upcoming paper now to help maximize its impact. If they'll be preparing press materials, please inform our press team within the next 48 hours. Your manuscript will remain under strict press embargo until 2 pm Eastern Time on the date of publication. For more information, please contact onepress@plos.org.

If we can help with anything else, please email us at customercare@plos.org.

Thank you for submitting your work to PLOS ONE and supporting open access.

Kind regards,

PLOS ONE Editorial Office Staff

on behalf of

Dr. Zhiwen Luo

Academic Editor

PLOS ONE
